# A whole-genome sequence and transcriptome perspective on HER2-positive breast cancers

**DOI:** 10.1038/ncomms12222

**Published:** 2016-07-13

**Authors:** Anthony Ferrari, Anne Vincent-Salomon, Xavier Pivot, Anne-Sophie Sertier, Emilie Thomas, Laurie Tonon, Sandrine Boyault, Eskeatnaf Mulugeta, Isabelle Treilleux, Gaëtan MacGrogan, Laurent Arnould, Janice Kielbassa, Vincent Le Texier, Hélène Blanché, Jean-François Deleuze, Jocelyne Jacquemier, Marie-Christine Mathieu, Frédérique Penault-Llorca, Frédéric Bibeau, Odette Mariani, Cécile Mannina, Jean-Yves Pierga, Olivier Trédan, Thomas Bachelot, Hervé Bonnefoi, Gilles Romieu, Pierre Fumoleau, Suzette Delaloge, Maria Rios, Jean-Marc Ferrero, Carole Tarpin, Catherine Bouteille, Fabien Calvo, Ivo Glynne Gut, Marta Gut, Sancha Martin, Serena Nik-Zainal, Michael R. Stratton, Iris Pauporté, Pierre Saintigny, Daniel Birnbaum, Alain Viari, Gilles Thomas

**Affiliations:** 1 Synergie Lyon Cancer, Plateforme de bioinformatique ‘Gilles Thomas' Centre Léon Bérard, 28 rue Laënnec, 69008 Lyon, France; 2 Institut Curie, PSL Research University, Département de Pathologie, INSERM U934, 26 rue d'Ulm, 75248 Paris, France; 3 Centre Hospitalier Universitaire de Minjoz, UMR INSERM 1098, Boulevard A. Fleming, Besançon 25000, France; 4 Plateforme de génomique des cancers, Centre Léon Bérard, 28 rue Laënnec, 69008 Lyon, France; 5 Institut Curie, UMR 3215 CNRS, Génétique et biologie du développement, Epigénèse et développement des mammifères, U934 Inserm, 26 rue d'Ulm, 75248 Paris, France; 6 Centre Léon Bérard, Département de Pathologie, 28 rue Laënnec, 69008 Lyon, France; 7 Département de Biopathologie, Unité Inserm U916, Institut Bergonié, 229 cours de l'Argonne, 33076 Bordeaux, France; 8 Centre Georges-François Leclerc et CRB Ferdinand Cabanne, 1 rue du Professeur Marion, Inserm U866-UBFC, 21000 Dijon, France; 9 Centre d'Etude du Polymorphisme Humain (CEPH), Fondation Jean Dausset, 27 rue Juliette Dodu, 75010 Paris, France; 10 Institut Paoli-Calmettes, Département de Pathologie, 232 Boulevard de Sainte-Marguerite, 13009 Marseille, France; 11 Institut Gustave Roussy, Comité de Pathologie Mammaire, 114 rue Edouard Vaillant, 94805 Villejuif, France; 12 Centre Jean Perrin, Département de Biopathologie, EA 4677 ERTICa, Université d'Auvergne, 58 rue Montalembert, 63000 Clermont-Ferrand, France; 13 Institut Régional du Cancer de Montpellier (ICM), Département de Pathologie, 208 Avenue des Apothicaires, 34298 Montpellier, France; 14 Institut Curie, PSL Research University, Service de Pathologie, Centre de Ressources Biologiques, BRIF BB-0033-00048, 26 rue d'Ulm, 75248 Paris, France; 15 Département de Pathologie, Institut Bergonié, 229 cours de l'Argonne, CS 61283, 33076 Bordeaux, France; 16 Institut Curie, PSL Research University, Département d'Oncologie Médicale, Université Paris Descartes, 26 rue d'Ulm, 75248 Paris, France; 17 Centre Léon Bérard, Département de Cancérologie Médicale, 28 rue Laënnec, 69008 Lyon, France; 18 Department of Medical Oncology, Institut Bergonié Unicancer, University of Bordeaux, INSERM U916, CIC1401, 229 cours de l'Argonne, CS 61283, 33076 Bordeaux, France; 19 Institut Régional du Cancer de Montpellier (ICM), Oncologie Sénologie, 208 Avenue des Apothicaires, 34298 Montpellier, France; 20 Centre Alexis Vautrin, Département d'Oncologie Médicale, 6 Avenue de Bourgogne, 54511 Vandoeuvre Les Nancy, France; 21 Centre Antoine Lacassagne, Département d'Oncologie Médicale, 33 Avenue de Valombrose, 06189 Nice, France; 22 Institut Paoli-Calmettes, Département d'Oncologie Médicale, 232 Boulevard de Sainte-Marguerite, 13009 Marseille, France; 23 Clinique Mutualiste de Bellevue, Chirurgie Gynécologique et Mammaire, 3 rue le Verrier, 42100 Saint-Etienne, France; 24 Institut Gustave Roussy, Cancer Core Europe, 39 rue Camille Desmoulins, Villejuif 94805, France; 25 CNAG-CRG, Centre for Genomic Regulation (CRG), C/Baldiri Reixac 4, 08028 Barcelona, Spain; 26 Universitat Pompeu Fabra, Plaça de la Mercè, 10, 08002 Barcelona, Spain; 27 Wellcome Trust Sanger Institute, Hinxton, Cambridge CB10 1SA, UK; 28 East Anglian Medical Genetics Service, Cambridge University Hospitals NHS Foundation Trust, Cambridge CB2 9NB, UK; 29 Institut National du Cancer, Département de Recherche Clinique, 52 Avenue Morizet, 92513 Boulogne-Billancourt, France; 30 INSERM U1052-CNRS 5286, Cancer Research Center of Lyon, F-69008 Lyon, France; 31 Université de Lyon, F-69622 Lyon, France; 32 Centre Léon Bérard, 28 rue Laënnec, 69008 Lyon, France; 33 Département d'Oncologie Moléculaire, Institut Paoli-Calmettes, Centre de Recherche en Cancérologie de Marseille, INSERM, CNRS, Aix-Marseille Université, 232 boulevard de Sainte-Marguerite, 13009 Marseille, France; 34 Equipe Erable, INRIA Grenoble-Rhône-Alpes, 655 Avenue de l'Europe, 38330 Montbonnot-Saint Martin, France

## Abstract

HER2-positive breast cancer has long proven to be a clinically distinct class of breast cancers for which several targeted therapies are now available. However, resistance to the treatment associated with specific gene expressions or mutations has been observed, revealing the underlying diversity of these cancers. Therefore, understanding the full extent of the HER2-positive disease heterogeneity still remains challenging. Here we carry out an in-depth genomic characterization of 64 HER2-positive breast tumour genomes that exhibit four subgroups, based on the expression data, with distinctive genomic features in terms of somatic mutations, copy-number changes or structural variations. The results suggest that, despite being clinically defined by a specific gene amplification, HER2-positive tumours melt into the whole luminal–basal breast cancer spectrum rather than standing apart. The results also lead to a refined *ERBB2* amplicon of 106 kb and show that several cases of amplifications are compatible with a breakage–fusion–bridge mechanism.

## Abstract (web summary)

Breast cancer is separated into multiple subtypes based on the expression of HER2 and hormone receptors. Here, the authors report the whole genome sequence of 64 HER2 positive tumours and show that these can be further separated into four groups with different gene expression profiles and genomic features.

## Graphical abstract

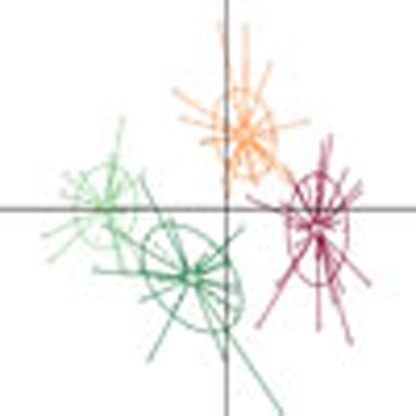

Two main classes of breast cancer (BC) are distinguished by the expression of hormone receptors (HR); namely oestrogen receptor (ER) and progesterone receptor (PR). HR-positive breast cancers have a better prognosis than HR-negative breast cancers. This classification is helpful in clinic but the classes are still greatly heterogeneous. Transcriptomic analyses have identified intrinsic molecular subtypes differing by their expression programs, including luminal A and B, basal and HER2-enriched subtypes^1^. Schematically, luminal breast cancers express HR while basal breast cancers do not. Because of this heterogeneity, due to various cell-of-origins and molecular alterations, the response of breast cancer patients to therapy is variable and difficult to predict^2^. Conversely, molecular alterations represent potential therapeutic targets. Amplification of the human epidermal growth factor receptor *ERBB2/HER2* gene, located in chromosomal region 17q12 occurs in around 15% of breast cancers and defines the category of clinical HER2-positive breast cancers. Overexpression of the ERBB2/HER2 protein kinase receptor has enabled patients with HER2-positive tumour to benefit from antibody-based (for example, trastuzumab) and anti-kinase-based (for example, lapatinib) therapies that target this receptor^3^,^4^,^5^,^6^,^7^. These therapies, in use these last 15 years, have completely changed the prognosis of HER2-positive tumours. However, HER2-positive breast cancers are heterogeneous. They may be included in the HER2-enriched or luminal molecular subtypes, depending on whether they express ER, and this has a consequence on their response to targeted therapies. Indeed, several clinical trials have pointed out the variability in efficacy of trastuzumab-containing regimens depending on ER and HER2 status of the tumours, suggesting heterogeneous biological characteristics^8^,^9^,^10^,^11^. Furthermore, the presence of additional molecular alterations, such as mutations of PI3 kinase or PTEN phosphatase, also has an impact on this response^12^,^13^,^14^,^15^,^16^. Thus, HER2-positive breast cancers vary in their genome alterations, gene expression programs, and cell-of-origin and this impacts on their microenvironment^17^,^18^, prognosis and response to treatment. A comprehensive analysis of HER2-positive tumour genomes should provide a definite basis for understanding this heterogeneity and the natural history of HER2-positive breast cancer and should help progress in the management of patients with HER2-positive tumours.

Here as part of the ICGC Breast Cancer Working Group effort, we have established gene expression profiles of 99 HER2-positive breast tumours and the complete genomes of a subset of 64 tumours were sequenced. A thorough analysis of this data set identifies four expression groups each of which exhibits distinctive genomic features such as mutations, copy-number variations (CNVs) or structural variations. Moreover, our results lead to a refined ERBB2 amplicon of 106 kb and show that some amplifications are compatible with a breakage–fusion–bridge (BFB) mechanism.

## Results

### Delineating four expression groups

A total of 289 HER2-positive (HER2+) BCs with frozen tumour samples identified from the French PHARE/SIGNAL programs^19^,^20^ were analysed. HER2, ER and PR statuses were defined according to ASCO/CAP guidelines^21^ (Methods). All cases were reviewed by breast pathologists and corresponding DNA and RNA samples were subjected to extensive quality controls (Methods). A total of 99 selected tumours were analysed for genome-wide expression profiles, out of which 64 tumours and matched normal DNA were subjected to whole-genome sequencing (WGS) (Supplementary Data 1).

An unsupervised hierarchical cluster analysis of the 99 HER2+ samples delineated four main groups of gene expression, herein referred to as A, B, C and D (Supplementary Fig. 1), validated on two external data sets^22^,^23^ (Methods). In addition, these 99 samples were assigned to PAM50 intrinsic subtypes^24^, using an external data set^25^ of 537 expression profiles that includes the whole spectrum of the BC disease and was hybridized on the same experimental platform to minimize technical biases (Methods).

Groups A and B were mostly composed of ER+ and luminal B tumours while ER- and HER2-enriched tumours were mostly observed in groups C and D (Table 1). Group D encompassed all (*n*=6) basal tumours. As expected, *ESR1*, *PGR* and *ERBB2* genes expression levels were tightly correlated with ER, PR and HER2 statuses, respectively, and decreased from groups A to D. Gene sets specific to the mammary stem cells (MaSCs), luminal progenitor (pLum) and luminal mature (mLum) populations^26^ were analysed, showing an increasing expression gradient from groups A to D for pLum and a corresponding decreasing gradient for mLum (Supplementary Fig. 2a–c).

### Distinctive genomic features of expression groups

**Figure 1:**
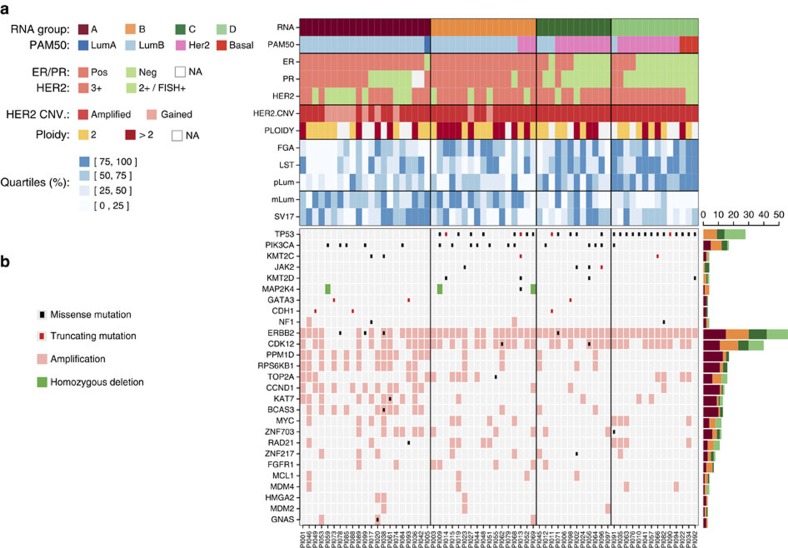
Summary of biological and genomic features of the 64 sequenced HER2+ tumours. (**a**) (From top to bottom) RNA expression groups; PAM50 subtypes; ER, PR and HER2 IHC statuses; HER2 CNV status; estimated ploidy; Fraction of genome altered (FGA) quartiles; number of large scale transitions (LST) quartiles; progenitor luminal gene signature (pLum) quartiles; mature luminal gene signature (mLum) quartiles; number of intrachromosomal SV in chromosome 17 (SV17) quartiles; (**b**) mutations, amplifications and homozygous deletions observed in a selected set of putative driver genes.

A total of 554,554 somatic mutations (549,003 single-nucleotide variations (SNVs) and 5,551 small insertions and deletions (indels)) were detected in the 64 tumours (Methods). The median mutation frequency was 1.95 per Mb (range 0.55–35.34 per Mb) in agreement with previous BC estimates^27^,^28^ (Supplementary Fig. 3). A total of 7,090 somatic mutations (1.3%) were located within coding exons, including 5,194 (73%) non-silent mutations in 3,845 different protein-coding genes. We identified 305 homozygous gene deletions (HD) (Methods) in 295 different genes. In total, 4,086 genes were altered (SNV or HD) in at least one tumour (Supplementary Data 2) and 52 genes in at least four tumours. Eight out of these 52 genes are known to be involved in cancer: *TP53*, *PIK3CA*, *JAK2*, *ATRX*, *MAP2K4*, *ERBB2*, *KMT2C* and *KMT2D*. *TP53* mutations were present in 28 (44%) tumours, including 21 missense mutations, 4 truncating mutations and 2 mutations altering splice junctions. *TP53* mutations were present in both ER+ and ER− cases, although more frequent in ER− than ER+ tumours (*P*=5 × 10^−3^, Fisher's exact test), and HER2-enriched and basal than luminal subtypes (*P*=5 × 10^−6^, Fisher's exact test) as previously observed^29^,^30^. Striking differences appeared when considering the four gene-expression groups (*P*= 7 × 10^−10^, Fisher's exact test). Group A was devoid of *TP53* mutations while all group D tumours were mutated (Fig. 1). *PIK3CA* displayed only missense mutations (18 mutations in 17 tumours), including 11 hotspot mutations (Supplementary Data 2). *MAP2K4* displayed HD in three ER+ cases and *GATA3* displayed three frameshifts. Six *ERBB2* missense mutations were observed in four tumours including two in the kinase domain^31^. To assess if modifications of potential *cis*-regulatory regions could also contribute to differences between the four expression groups, we studied somatic SNVs in these regions by looking at a combination of histone marks enriched in regulatory region and FANTOM5 enhancer and promoter annotation^32^ (Methods). An under-representation of SNVs located in H3K4me1 and H3K27ac enhancers was observed in ER+ tumours (*P*=8 × 10^−3^, Mann–Whitney *U*-test) and in group A (*P*=8 × 10^−3^, Kruskal–Wallis test; *P*=6 × 10^−4^, Mann–Whitney *U*-test, A versus BCD) (Supplementary Fig. 2d,e).

Somatic CNVs were derived from WGS for the 64 sequenced tumours (Methods). CNV frequency profiles (Supplementary Fig. 4) were in good agreement with published profiles^33^,^34^ of HER2+ tumours including the *ERBB2* amplicon as well as recurrent CNVs found in BCs. Some CNVs were group specific: gains of 2p and 2q chromosomal arms were more frequent in group D (*P*=1 × 10^−3^ and 3 × 10^−3^, respectively, Fisher's exact test), while loss of 11q was more frequent (*P*=5 × 10^−3^, Fisher's exact test, FDR corrected) and loss of 14q was less frequent (*P*=1 × 10^−2^, Fisher's exact test, FDR corrected) in group A. The median fraction of the genome altered (FGA) was 59%, a number somewhat higher than previously reported by using array-CGH^33^ (34%). FGA was lower in group A than in the three other groups (*P*=2 × 10^−2^, Kruskal–Wallis test; *P*=2 × 10^−3^, Mann–Whitney *U*-test, A versus BCD) (Supplementary Fig. 2f).

As already reported^33^, other regions from 17q were found to be amplified including the 17q21.32-17q21.33 region that harbours *SPOP* (amplified in 14% of the tumours) and *KAT7* (20%) genes as well as the 17q23.1-17q24.3 region that harbours *RPS6KB1* (25%), *PPM1D* (27%), *BCAS3* (20%) and *DDX5* (16%) genes. These two regions appeared more frequently amplified in group A (Supplementary Fig. 5). High-level gene amplifications were observed on chromosomes 8, 11 and 20 in more than 10% of the patients and on chromosomes 1, 6 and 12 in 5–10% of the patients (Supplementary Fig. 6). These regions included several known or putative oncogenes (Supplementary Data 3): 8p11.23 (*ZNF703* (17%), *WHSC1L1* (11%), *FGFR1* (11%) and *PPAPDC1B* (9%); 8q23.1 (*RSPO2* (16%) and *EIF3E* (14%)); 8q24.11 (*RAD21* (16%)); 8q24.21 (*MYC* (19%)); 11q13.3 (*CCND1* (22%)); 20q13.2 (*ZNF217* (11%)). Amplification of *PPM1D* and *CCND1* was more frequent in group A than in the other groups (*P*=2 × 10^−3^ and 4 × 10^−3^, respectively; Fisher's exact test, FDR corrected).

A total of 133 firestorms^35^ (Methods) were detected in 27 different chromosomal arms in 58 patients (90%). Although firestorms were observed at least once in all chromosomal arms, they were more frequent in 17q (*n*=41 tumours); 8q (*n*=18) and 11q (*n*=10).

A total of 24,203 somatic structural variations (SVs) were detected (Methods), with a median of 327 per sample (range 132–952) in agreement with the reported values^36^. The majority (75%) of these variants were intra-chromosomal (Supplementary Fig. 7) and composed of 7,438 (41%) inversions, 5,889 (33%) deletions and 4,731 (26%) duplications. The numbers of intra- and inter-chromosomal SVs were correlated (Kendall tau=0.46, *P*=7 × 10^−8^, Kendall's test). Intra-chromosomal SVs were more frequent on chromosome 17 with a median of 30.5 SVs than on all other chromosomes (median of 6.0; *P*<1 × 10^−10^, Mann—Whitney *U*-test). Although no association was observed between the number of SVs on chromosome 17 and ER status, RNA groups A and C displayed more intra-chromosomal SVs on chromosome 17 than groups B and D (*P*=4 × 10^−3^, Mann—Whitney *U*-test for AC versus BD; Supplementary Fig. 8). The most frequent inter-chromosomal rearrangements were observed between pairs of chromosomes (17 and {8, 11}); (1 and {2, 3, 5, 6, 8}) and (8 and 6). ER− tumours displayed more scattered inter-chromosomal rearrangements than ER+ tumours. More specifically, RNA group A displayed focused rearrangements, mostly limited to (17 and {8, 11}) and (1 and {2, 6}), while RNA group D displayed the most scattered pattern (Supplementary Fig. 9). The number of inter-chromosomal rearrangements in a sample was associated with the presence of a *TP53* mutation (*P*=5 × 10^−2^, Mann–Whitney *U*-test). The homologous recombination deficiency, measured by computing a *BRCAness* score as previously reported^37^ (Methods), was increasing from RNA groups A through D (Supplementary Fig. 2g).

### The ERBB2 amplicon

Using precise CNV levels as well as SV breakpoints from WGS data allowed us to delineate precisely the 17q12 *ERBB2* amplicon as a 106-kbp region (chr17:37818020-37924454, GRCh37 build) that was amplified or gained in all the samples, thus refining the 248-kbp region (chr17:37725640-37973561) previously determined using array-CGH^33^. The *ERBB2* amplicon included six genes: *TCAP*, *PNMT*, *PGAP3*, *ERBB2*, *MIEN1* and *GRB7*.

**Figure 2:**
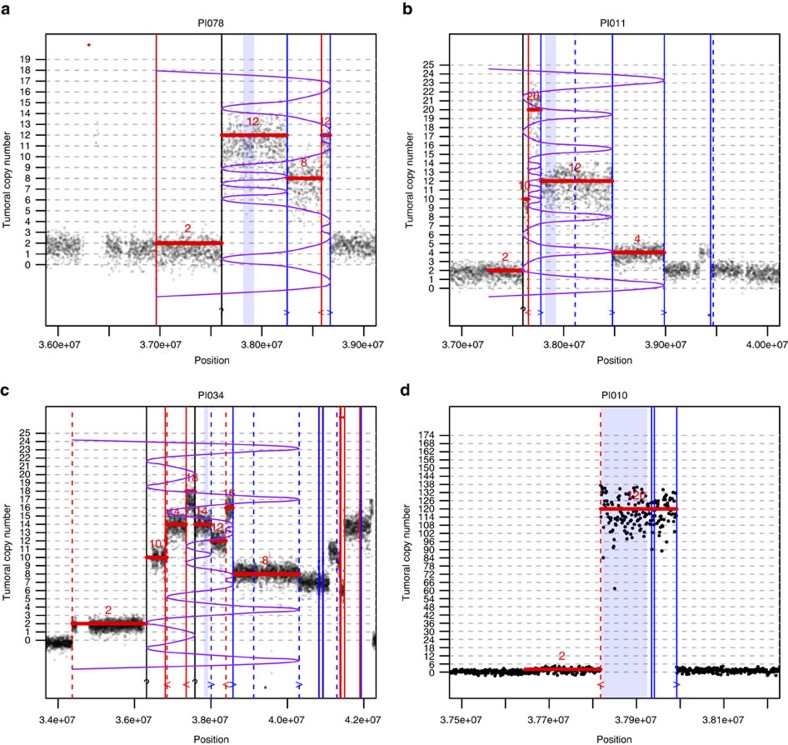
Examples of CNV patterns in the ERBB2 amplicon. *X* axis: position on chromosome 17, the minimal region defining the ERBB2 amplicon is indicated by the blue shaded box. *Y* axis: tumoral integer copy number, computed in 1 kb binned read counts (Methods). Horizontal red segments indicate the closest sequence consistent with a breakage−fusion−bridge (BFB) fold and the purple curved line represents the corresponding folding pattern. Vertical lines indicate the location of detected breakpoints from discordant and clipped read pairs (Methods); plain lines correspond to intra-chromosomal events and dashed lines to inter-chromosomal events; line colour (and glyph on the bottom) correspond to the clipped reads orientation, red (<): reads clipped to the left, blue (>) reads clipped to the right. If the predicted BFB folding pattern is correct, clipped reads orientation should correspond to a left (resp. right) fold in the purple line. Black lines correspond to missed breakpoints, which (approximate) position could be inferred from a copy-number change. (**a**) A BFB consistent sequence composed of intra-chromosomal events (plain lines) only. (**b**) Same as previous with additional inter-chromosomal events (dashed lines) not involved in BFB. (**c**) A BFB consistent sequence composed of both intra- and inter-chromosomal events. The presence of inter-chromosomal events suggests that the amplification process may involve other chromosome(s). (**d**) Example of a focal amplification, unlikely to occur by a BFB mechanism, suggesting that other mechanisms (double minutes) may be involved.

In several tumours, the CNV patterns as well as the clipped read orientations were consistent with a BFB mechanism. BFB is a DNA amplification mechanism that has been discovered in maize in the late 30s (ref. 38) but later evidenced in tumours^39^,^40^,^41^. It occurs when a chromosome undergoes a double-strand break (Supplementary Fig. 10a) followed by the erroneous fusion of the two loose ends of the sister chromatids during replication. This results in the formation of a bridged dicentric chromosome that is torn apart during the next anaphase (Supplementary Fig. 10b), inducing a further breakage that will repeat the process. At each cycle, stretches of DNA close to the breakpoints are duplicated head to head leading to the exponential accumulation of palindromic sequences in the region containing breakpoints (Supplementary Fig. 10c). When mapped to the reference chromosome, these sequences will adopt a typical fold-back structure that is the hallmark of BFB. The process stops when the loose telomere is capped or fused to another chromosome to produce a translocation. In terms of sequence data there are two main hallmarks of BFB process. First the copy-number pattern follows some specific sequences^42^,^43^ (Supplementary Fig. 10d), that is, not all possible discrete values are allowed. Second, when using paired-end sequencing, read pairs with discordant orientations mark the vicinity of breakpoints. In addition, reads spanning breakpoints may be clipped to the right or to the left depending on the direction of the fold. Therefore, the orientation of discordant pairs and clipped reads provide additional clues to characterize a BFB fold. Figure 2a–c gives three examples of such patterns, consistent with a BFB mechanism for *ERBB2* amplification^39^. In some cases (Fig. 2c) several breakpoints were associated with inter-chromosomal events, suggesting that the amplification may have taken place on other chromosomes. We also observed patterns that are very unlikely to have occurred by a BFB process, such as the focal amplification depicted in Fig. 2d, suggesting that other mechanisms may also be involved in *ERBB2* gene amplification such as the formation of double-minutes chromosomes^44^. Finally, some intricate fold patterns may also suggest that several mechanisms may sometimes combine at the same locus.

## Discussion

**Figure 3:**
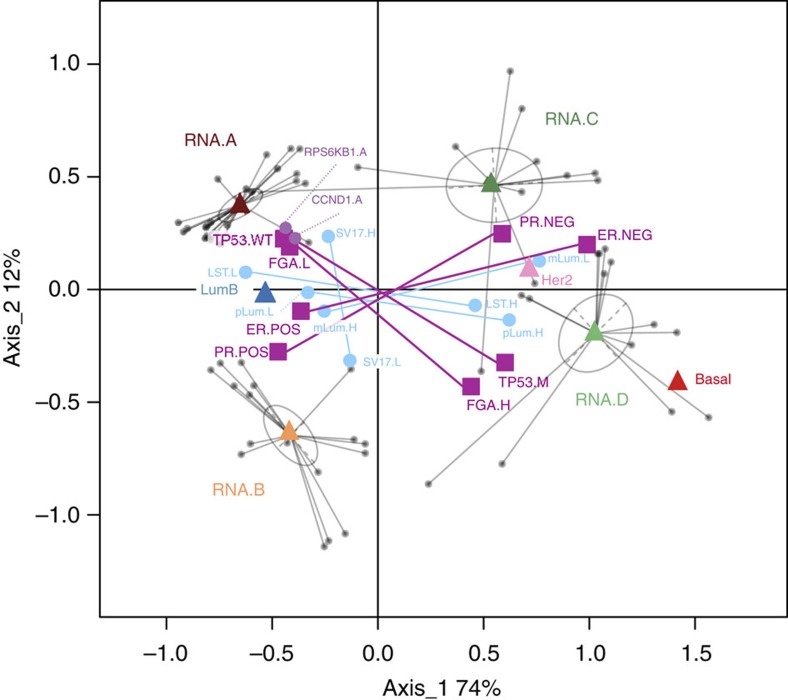
Multiple correspondence analysis of selected biological and genomic variables. Patients are represented by small grey dots and variables (categories) are represented by coloured squares, triangles and large dots. Categories indicated by triangles and squares were used for MCA analysis whereas categories indicated by larger dots were just projected on the resulting map. This map has a simple geometrical interpretation: a category is plotted at the centre of gravity of the patient points for those patients that choose that category (conversely, at a scaling factor, patient points are located at the centre of gravity of categories they choose). As an example, patients points have been linked to the RNA group they belong to (grey ellipses). Therefore the proximity of two categories suggests that they are chosen by a similar set of patients. Categories labels are as follows: RNA groups (RNA.A, RNA.B, RNA.C and RNA.D); PAM50 subtypes (LumB, Her2, Basal); ER status (ER.POS, ER.NEG), PR status (PR.POS, PR.NEG); *TP53* mutations: (TP53.M, TP53.WT); fraction of genome altered (FGA.L (lower quartile), FGA.H (upper quartile)); number of interchromosomal SVs in chromosome 17 (SV17.L (lower quartile), SV17.H (upper quartile)); Number of Large Scale Transitions (BRCAness score) (LST.L (lower quartile), LST.H (upper quartile)); progenitor luminal signature score (pLum.L (lower quartile), pLum.H (upper quartile)); mature luminal signature score (mLum.L (lower quartile), mLum.H (upper quartile)); for clarity, interquartile points are not plotted.

We used gene expression as an operational basis to classify HER2+ tumours into four groups that were further characterized in terms of interdependent genomic variables. A synoptic outline of the dependencies between these variables, provided by multiple correspondence analysis (MCA) (Methods) is shown on Fig. 3. Groups A and B were ER+ tumours, harboured genomic alterations observed in luminal BCs and were close to the luminal B intrinsic subtype. Main features of group A (left part of Fig. 3) were the absence of *TP53* mutations, low FGA, higher number of SVs on chromosome 17 and specific amplifications in *CCND1* (11q13) and *RPS6KB1* (17q23) regions. In contrast, tumours in groups C and D were mostly ER− and close to the HER2-enriched intrinsic subtype. *TP53* mutations were frequent in these groups and analysis of genome breakages showed higher FGA and some pattern of BRCAness. Moreover, analysis of the mLum and pLum signatures suggested that A and B tumours derive from differentiated mature luminal cells, while C and D derive from luminal progenitors, like basal tumours. Altogether, these results suggest that HER2+ BCs do not *per se* represent an actual intrinsic subtype but, instead, are distributed along the whole BC spectrum, from ER+ luminal to ER− basal phenotype, with genome alterations in accordance to these phenotypes and are incidentally characterized by a specific gene amplification.

Although patients with HER2+ BC benefit from HER2-targeted therapies, the response is highly variable and a significant number of patients display primary or secondary resistance. Heterogeneity of these cancers may explain the extent of this variability. Thus, how important the molecular and cell-of-origin definition of HER2 tumours may be to understand BC oncogenesis, the prime interest of the medical community lies in the therapeutic opportunity provided by the identification of homogeneous subgroups and a better understanding of the genetic mechanisms involved. Clinical follow-up of our cohort of HER2+ tumours will determine the importance of splitting HER2+ BCs in A to D groups.

## Methods

### Patient samples and ethical approval

A total of 289 female patients diagnosed with HER2-positive breast cancer were recruited through the French PHARE/SIGNAL trial^19^,^20^. The trial was sponsored by the French National Cancer Institute (INCa), approved by the Central Ethical Committee (Comité de Protection des Personnes, CHU Besançon) on 15 May 2006. It was done in compliance with the principles of Good Clinical Practice and the Declaration of Helsinki and registered at ClinicalTrials.gov, number NCT00381901. Patients were eligible if they were over 18 years of age with histologically confirmed invasive early breast cancer with HER2 overexpression and had provided signed informed consent. For each patient, tumour tissue as well as matched blood samples were collected. Tumours were snap-frozen in liquid nitrogen upon surgical removal after pathologist's review and were stored in the corresponding hospital's biological resources center. The clinical HER2 status was assessed in accordance with the ASCO/CAP guidelines^21^. HER2 protein expression was scored by performing immunohistochemistry (IHC) on a tumour section from FFPE blocks for all patients. Cases displaying a 3+ score were considered positive and cases only displaying a 2+ score were tagged as equivocal. In equivocal cases, HER2 gene amplification status was further determined using either fluorescence *in situ* hybridization (FISH) or chromogenic *in situ* hybridization (CISH) and only those showing an HER2 gene amplification were then considered positive. ER and PR statuses were also established using IHC. Breast pathologists reviewed all cases for ER, PR and HER2 status. Corresponding pathological, clinical and follow-up data were obtained from the INCa PHARE/SIGNAL clinical database.

### Sample extraction

Samples had full face sectioning performed in with Tissue-Tek optimal cutting temperature (O.C.T) compound to estimate the percentage of malignant epithelial nuclei in the sample relative to stromal nuclei. Macrodissection was performed if required to excise areas of non-malignant tissue. DNA and RNA were then extracted from the same sample. Total genomic DNA was extracted with phenol-chloroform after proteinase K digestion, followed by the precipitation of nucleic acids in ethanol. DNA was quantified using Nanodrop spectrophotometer ND-1000 (ThermoScientific, Wilmington, USA) and Qubit BR DNA assay (Invitrogen). RNA was also extracted using the miRNeasy miniKit (Qiagen) in accordance to the manufacturer's protocol. RNA was quantified using Nanodrop spectrophotometer ND-1000 and the purity and integrity were assessed by the Agilent 2100 Bioanalyzer and RNA 6000 Nano Labchip Kit (Agilent Biotechnologies, Palo Alto, CA, USA). All matched peripheral bloods have been centralized and then extracted using the salting-out method with a Qiagen Autopure LS (Courtaboeuf, France) in the Fondation Jean Dausset CEPH laboratory. To confirm the matching between tumour and blood DNA issued from a same patient, the AmpFLSTR Identifiler PCR Amplification Kit (Life Technologies) has been used.

### Selection of high-quality HER2-positive samples

A total of 289 tumour samples (and matched blood samples) were processed as described in the previous section. A special attention was then paid to the selection of a high-quality subset of these samples for further analysis. Proportion of tumour cells were estimated on frozen tumour sections by pathologists and only those estimated with at least 50% tumour cells were kept. All DNA and RNA samples were subjected to quality controls (RNA integrity number ≥7; DNA integrity checked on agarose gel) leaving a subset of 131 samples. The tumour DNAs of these 131 patients were hybridized on Illumina OmniExpress arrays to establish the genomic profile of each tumour. These genomic profiles were used to control the presence of the ERBB2 gene amplification and to obtain another estimation of the tumour purity (see SNP array processing section below for details). A missing ERBB2 amplification/gain or a very low estimated purity caused the sample to be discarded. At the end of the process, 99 samples (hereafter called the INCa-HER2+ data set) met the required quality criteria, out of which 64 were subjected to WGS.

### Gene expression array processing and quality control

Tumour RNA samples were analysed for expression profiling on Affymetrix U133 Plus 2.0 GeneChip. Quality control was asserted by using R package affyPLM^45^. Raw feature data were normalized using robust multi-array average^46^ method in R package affy^47^. Probe sets corresponding to control genes were filtered out.

### PAM50 subtypes classification

Each tumour gene expression profile from the INCa-HER2+ data set was assigned to a PAM50 breast molecular subtype^24^. The PAM50 classifier was built using a training cohort, which aims at capturing the major breast cancer types in the general population. As the INCa-HER2+ data set was exclusively composed of HER2+ samples, the underlying distribution of expression profiles was likely to be not representative of the whole spectrum of breast cancers expression profiles^48^. To overcome this difficulty, we collected 537 Affymetrix expression profiling of all types of breast cancers from the Carte d'Identité des Tumeurs (CIT) project^25^ from the French Ligue Nationale Contre le Cancer. This cohort was hybridized on the same microarray and on the same experimental platform (at IGBMC Strasbourg) than the INCa-HER2+ data set, thus minimizing technical biases. The CIT data set is available on ArrayExpress under accession number E-MTAB-365. First, all expression profiles from CIT and INCa-HER2+ data sets were normalized together using the robust multi-array average method^46^ as above. Then, to maintain the relative proportions of the PAM50 subtypes, each of the 99 INCa-HER2+ samples was included one by one into the CIT data set and assigned to a PAM50 subtype using the R package genefu^49^ v1.12.0 with robust scaling of the gene expressions centroids.

### Unsupervised clustering of transcriptomic array profiles

The clustering of transcriptomic array profiles was performed in two steps. The first step aims at selecting probesets carrying the most differential expression across the data set. This was done by using two criteria: (a) for each probeset, we tested whether its variance across samples was different from median of the variances of all the probesets. The statistics and criterion used were the same as in the filtering tool of BRB ArrayTools software^50^, where the variance to median ratio is compared with a percentile of the chi-square distribution. Only probesets satisfying a *P* value of this variance test <10^−3^ were kept. (b) In addition, probesets were ordered by relative s.d. and the top 5‰ percentile was retained. After this step, we were left with 274 probesets corresponding to 196 known genes. The second step was an agglomerative hierarchical clustering using Pearson correlation as a similarity measure and the Ward's minimum variance linkage method.

### Validation of RNA groups on TCGA and Metabric HER2+ data sets

Two publicly available expression profiling data sets were collected for validation purpose. The first one was the Breast Invasive Carcinoma (BRCA) collection of 1,098 tumours from TCGA^22^. We selected the subset of 114 samples defined as HER2+ in the original paper, out of which 75 had associated Agilent G4502A microarray expression data. The second validation set came from the Metabric breast cancer collection^23^. We selected a subset of 122 HER2-amplified tumours with IHC status equal to 2+ or 3+ and copy number of ERBB2 locus gained according to SNP6 array (no IHC-FISH status was available in this data set). Raw Illumina HT-12-v3 expression profiling data were obtained after data access authorization and normalized using R package beadarray^51^. To allow the comparison between expression data from different array technologies, we reduced the RNA group signature defined with probesets to genes by using the best genes according to JetSet^52^. This resulted in 196 genes out of which 162 and 180 were defined in TCGA and Metabric data respectively.

We used two different and complementary methods for validation on both sets. The first method is a single sample predictor (SSP) method. First the centroid of each RNA group (labelled A, B, C, D) was computed on the centred-reduced INCa data set. Then, for each single external sample (that is, from TCGA or Metabric), we computed the Spearman rho correlation coefficient with each of these centroids and the sample was assigned to the RNA group with the largest correlation coefficient or to no group (O) if the correlation coefficient was <0.1. Independently, the whole external data set was clustered into four clusters (labelled 1, 2, 3, 4) using the same agglomerative hierarchical clustering method as before. The stability of the RNA groups in the external data set was then evaluated by examination of the (A, B, C, D, O) × (1, 2, 3, 4) contingency table and practically measured by the fraction of the most abundant RNA label in each cluster (that is, 100% if all clusters are composed of a single RNA group and, about 25% if RNA groups are spread randomly across clusters). The second method (joined) consisted in merging the independently centred-reduced INCa and external data sets. This joined set was then clustered into four clusters (labelled 1, 2, 3, 4) using the same agglomerative hierarchical clustering method as before and the stability of the RNA groups was then evaluated by examination of the (A, B, C, D) × (1, 2, 3, 4) contingency table of the data reduced to the INCa-HER2 subset (that is, for which the actual RNA group labels are known). A RNA group label can further be assigned to each of the four clusters (by considering the majority label in the INCa-HER2 subset) and, eventually, to each external sample. Finally, for both methods, clinical and biological metadata (namely ER status, PAM50 subtypes and P53 mutation status) per RNA group were also compared.

Results are shown in Supplementary Figs 11−15. Supplementary Fig. 11 displays the RNA groups obtained on the original INCa-HER2 data set using all of the 196 probesets as the reference. Supplementary Figs 12 and 13 (resp. 14 and 15) displays the results obtained on the TCGA (resp. Metabric) data set for both SSP and joined methods. The two methods gave similar results, with 76% (TCGA) and 81% (Metabric) of samples classified in the same RNA group by both methods. The overall stability of RNA groups is good (80% (SSP), 75% (joined) for TCGA and 66% (SSP), 73% (joined) for Metabric). Groups A and D appeared more stable than B and C. Finally, in terms of metadata, the same general features were observed for the three data sets: (a) over-representation of ER+ (resp. ER−) in groups A and B (resp. C and D); over-representation of luminal B (resp. HER2-enriched and basal) in groups A and B (resp. C and D). As for *TP53* mutations, the situation is less clear-cut: the under-representation of *TP53* mutations in group A is observed both for INCa and TCGA data sets but is not significant for the Metabric data set. However it should be pointed out that (a) this data set exhibits a lot of *TP53* undetermined cases (51%) and (b) for determined cases the fraction of *TP53* mutated cases is unusually low (28% versus 44% and 47%, respectively in INCa and TCGA data sets).

Finally, all cases in the present study were included in the PHARE/SIGNAL cohorts (NCT00381901-RECF1098, www.e-cancer.fr) with a median follow-up at 58 months (interquartile range 46.5–62.5). In this smaller subset and on this period, recurrence events were very limited (4 out of all 99 cases) and the relationship between RNA groups and outcome could not be analysed.

### Gene expression signature projection

Gene signatures for mammary stem cell, progenitor and mature luminal^26^ were projected by using the single sample extension of GSEA^53^ where gene expression values for each single sample were rank-normalized, and an enrichment score was produced using the empirical cumulative distribution functions of the genes in the signature and in the remaining genes.

### SNP array processing

Illumina Omni1 Quad and OmniExpress SNP arrays quality control and normalization was performed using GenomeStudio Genotyping Module. A supplementary normalization step was applied using the tQN algorithm^54^. Allelic CNVs were analysed using GAP^55^ and ASCAT 2.0 (ref. 56). Tumour purity was estimated using both approaches, with a good correlation, although GAP estimates were found to be always above ASCAT estimates (Supplementary Fig. 16a). This allowed correcting these estimates by using their geometric mean (Supplementary Fig. 16b). Estimates provided by pathologists were systematically above all others and were not used for the final estimation. Ploidy was estimated by using DNA indexes provided by the two methods and few discrepancies were resolved by nearest neighbours clustering (Supplementary Fig. 17). It should be noted that although DNA index of diploid tumours was centred around 1.1, DNA index of aneuploid tumours was centred on 1.7, therefore these tumours were probably resulting from a tetraploidisation event followed by chromosomal losses.

### Sequencing and genome alignment

WGS was performed on 64 tumours and matched normal DNA from the same individuals. Tumour and normal DNAs were sequenced to >45-fold and >30-fold coverage, respectively. Illumina HiSeq2000/ HiSeq2500 genome analysers and Illumina paired-end sequencing protocols were used for all samples, read lengths ranging from 100 to 126 base pairs. Paired-end reads were aligned to the human genome (*GRCh37*) using the BWA aligner^57^. Alignments were refined using GATK^58^ and Picard (http://broadinstitute.github.io/picard/) software suites. Duplicates were removed from the sample BAM files for further analysis. Raw and mapped sequences from all produced HiSeq lanes were checked using in-house pipelines that collect a set of important metrics reflecting the overall quality of the sequencing data. Lanes showing poor quality were manually discarded.

### SNV variant calling

Somatic SNVs were called using MuTect^59^ v1.1.5 part of the GATK suite. To improve performance, data from dbSNP Build 132 and COSMIC v65 (http://www.sanger.ac.uk/genetics/CGP/cosmic/) were supplied as parameters to MuTect. On top of this, we used in-house post-processing filters to improve the specificity of mutation calls. These filters include adjustments on strand bias, local coverage, position of alternate allele within the read, mapping quality, repeated regions. Moreover, a panel of normal genomes, generated on the same sequencing technology, was used to dismiss systematic sequencing errors and/or low frequency polymorphisms. SNV that passed all these filters were then annotated using the variant effect predictor^60^ tool v75.

### Mutations in *cis*-regulatory regions

Annotation of somatic variants that are located in *cis*-regulatory regions was performed using OncoCis^32^, based on human mammary epithelial cell epigenomic data sets. Annotated somatic variants were further processed to classify those in potential promoter or enhancer regions. Somatic SNVs in potential promoter regions were extracted if they localize around H3K4me3 histone marks. Somatic variants in potential enhancer regions were extracted if they localize around H3K4me1 or H3K27ac histone marks. In addition, mutations were further annotated using FANTOM5 predictions^61^, which uses cap analysis of gene expression to detect potentially active transcription from promotor or enhancer regions. In both cases (promotors and enhancers regions) we computed, per patient, the fraction of somatic SNVs located in these regions (that is, number of SNVs in regions divided by the total number of SNVs in patient).

### Copy-number analysis

The analysis of CNVs from whole-genome sequence data was performed in three steps. First, after reads alignment using the Burrows-Wheeler alignment tool (BWA), raw read counts at each genomic position were corrected by GC content using the approach described by Benjamini and Speed^62^. We slightly improved the construction of the empirical dependency model by using only raw counts in mappable^63^ regions, by sampling positions (10^7^) over the whole genome and ignoring positions with extreme read counts. In a second step, the GC-corrected relative read counts (*rRC*, defined as raw counts divided by predicted counts) were computed within 1 kb windows in mappable regions and smoothed along the chromosomes (Kalman filter) to get a well-resolved distribution of the observed levels. This distribution was used to evaluate the tumour contamination by normal DNA by fitting a sum of evenly spaced Gaussian peaks (the separation between two consecutive peaks being equal to 1/(*Q*+2*α*(1−*α*)); with *α*∈[0,1] the contamination and *Q* the tumour mean ploidy). Finally, a univariate Gaussian Hidden Markov Model (HMM, R package RHmm) was build, with parameters (levels and variance) inferred from the previous step. This HMM was then used to segment the GC-corrected relative read counts signal along the chromosomes. The resulting segment levels are expressed as a relative tumoral CN (*rCN*) corresponding to the states of the HMM, that is, *rCN*=1, the reference CN state, corresponds to the tumour mean ploidy (for example, 2 for a diploid tumour), 0.5 corresponds to the loss of half of the copies of the reference CN state (that is, hemizygous state for a diploid tumour). These relative tumoral CN segments were further used to define the gain/loss status of regions, using the following scale: *rCN*>3: amplification; 3≥*rCN*>1: gain; *rCN*<1: loss; *rCN*=0: homozygous deletion.

### Firestorms and large-scale transitions

A chromosome arm of high genomic complexity and which harbours multiple closely spaced amplicons is said to be in firestorm^35^. Using the CNV profiles computed from WGS data (see above) on each tumour, a chromosome arm was claimed in state of *firestorm* if it had at least 20 genomic segments reaching at least 10 different levels of copy number and if, among those segments, at least 5 were amplifications (rCN>3).

Large-scale state transitions (LST) were defined^37^, as chromosomal break between adjacent regions of at least 10 Mb each. As suggested^37^, the number of LSTs in the tumour genome was estimated for each chromosome arm independently (not accounting for centromeric or unmappable regions breaks) and after filtering and smoothing of all variations <3 Mb.

### Structural variants calling

Somatic SVs were identified by using two complementary signals from read alignments: (a) discordant pair mapping (wrong read orientations or insert-size larger than expected) and (b) soft-clipping (first or last bases of read unmapped) that allows to resolve SV breakpoints at the base pair. Each SV candidate was defined by a cluster of discordant pairs and one or two clusters of soft-clipped reads. The discordant pairs cluster defined two associated regions (possibly on different chromosomes) and the soft-clipped reads cluster(s), located in these regions, specified the potential SV breakpoint positions. We further checked that the soft-clipped bases at each SV breakpoint were correctly aligned in the neighbourhood of the associated region. Events were then classified as germline or somatic depending on their presence in the matched normal set of events. Somatic SVs were further filtered according to several criteria: at least 2 discordant read pairs per cluster; at least 2 soft-clipped reads per cluster; at least 15 aligned clipped bases and at least 1 breakpoint should be located in a mappable region^63^. Structural variants were then classified as intra-chromosomal event (deletion, duplication, inversion) or inter-chromosomal (breakpoints on different chromosomes) according to discordant read pairs type.

### BFB amplification analysis

We used two independent sources of information for testing the BFB amplification mechanism for the ERBB2 amplicon: the CNV patterns and discordant read pairs and clipped reads orientation at breakpoints. The main hallmark of BFB is that the CNV pattern follows some specific sequences^43^,^64^. A technical difficulty is that this requires the determination of tumoral absolute integer copy number (*aCN*), whereas NGS data primarily provides relative read counts (*rRC*, see section ‘Copy-number analysis'); *aCN* linearly relates to *rRC* as a function of the contamination by normal DNA and the mean ploidy of the tumour. So we first determined precisely these two quantities by plotting and fitting *rRC* versus allelic frequencies of SNPs over all the genome. We then segmented the *aCN* profile by using the same HMM procedure as before (section ‘Copy-number analysis'). Finally, we used the algorithm developed by Zakov and Bafna^42^ to check if the observed *aCN* sequences were compatible with a BFB sequence and/or to look for the longest compatible sequence. This is basically the same approach used by Greenman *et al.*^65^. Another possible approach is to estimate the rearrangement process and copy numbers simultaneously^43^. However, the later approach has a risk of overfitting the data, especially for short sequences. Therefore, except for one case (PI034), we did not readjust the predicted *aCN* levels to fit with a BFB sequence. In the case of PI034, we had to shift the read count signal by a constant value (2) to get a valid BFB sequence. This may be explained by the presence of two copies of the homologous chromosome 17 in this tumour. Beyond checking for the validity of a putative BFB sequence, the Zakov and Bafna algorithm^42^ also provides the corresponding folding pattern (visually represented by the purple curved line on illustrations provided in Fig. 2a−c). This allows for an additional and independent confirmation of the BFB fold. To this purpose we superimposed the location of somatic SV breakpoints (see section ‘Structural variant calling') to the CN plot. Of course the curve should ideally fold at identified breakpoints, but, more importantly, the folding pattern should fit with the clipped reads orientation. More precisely, when the fold occurs to the left side, the reads should be clipped on their right (3') side (that is, they align on their left part) and, conversely, when the fold occurs to the right side, the reads should be clipped on their left side. Therefore by simply colouring the breakpoint locations, we could visually check if the clipped reads orientation was compatible with the proposed BFB folding pattern. In all of the cases we examined, this test was always successful therefore providing a strong support in favour of the hypothesis that the folding pattern was indeed generated by a BFB mechanism.

### Multiple correspondence analysis

MCA is a multivariate statistical approach suited to the exploratory analysis of nominal categorical data^66^. It is an extension of correspondence analysis when more than two variables are involved. It can also be considered as an adaptation of principal component analysis (PCA) to nominal (instead of quantitative) data, using the chi-square (instead of euclidean) metric. Briefly, categorical variables are first encoded into boolean complete disjonctive form. For instance the RNA group variable has four categories (A, B, C, D), a patient in category A is therefore encoded as RNA.A=1, RNA.B=0, RNA.C=0, RNA.D=0. Then this complete disjunctive table is submitted to standard correspondence analysis using the ADE4 R package^67^ with Benzécri correction^68^ of the eigenvalues. Quantitative variables (for example, FGA) should be first encoded to categorical. To this purpose, we use the upper, lower and interquartile categories. The purpose of MCA is to construct a joint map of patients and variable categories in such a way that a patient is close to a category it is in, and far from the categories it is not in (conversely a category is close to the patients that have it and far from patients that do not have it). This map has a simple geometrical interpretation^69^, thanks to the centroid principle: MCA plots a category point at the centre of gravity of the patient points for those patients that choose that category (conversely, at a scaling factor, patient points are located at the centre of gravity of categories they choose). Finally, it should be pointed out that beside variables categories used for the analysis, it is also possible to project additional categories (or patients) onto the map. The projected category is simply located at the centre of gravity of those patients that choose this category.

### Data availability

Raw data have been uploaded to the European Genome-phenome Archive (EGA; http://www.ebi.ac.uk/ega) under the overarching study accession number EGAS00001001431. This study includes all data from whole-genome sequencing, genotyping arrays and gene expression arrays used in this work. Access to whole-genome sequences and genotyping arrays is subjected to the ICGC data access authorization (DAC: EGAC00001000010 and Policy: EGAP00001000037). The CIT data set is available on ArrayExpress (http://www.ebi.ac.uk/arrayexpress) under accession number E-MTAB-365. METABRIC expression data sets are available at EGA under study accession number EGAS00000000083. TCGA expression data set is available on the TCGA data portal (https://tcga-data.nci.nih.gov/docs/publications/brca_2012). The remaining data are contained within the Article or Supplementary Information files, or available from the authors upon request.

## Additional information

**How to cite this article:** Ferrari, A. *et al.* A whole-genome sequence and transcriptome perspective on HER2-positive breast cancers. *Nat. Commun.* 7:12222 doi: 10.1038/ncomms12222 (2016).

## Supplementary Material

Supplementary Information Supplementary Figures 1-17

Supplementary Data 1 Characterisation of the 99 samples of the HER2+ cohort. Sequencing, clinical, anatomo-pathological and biological characterisation.

Supplementary Data 2 List of somatic SNVs in genes.

Supplementary Data 3 Frequently amplified genes.

## Figures and Tables

**Table 1:** Associations between RNA expression groups and biological characteristics for the 99 samples of the INCa-HER2+ data set.

	**RNA group**				**Total**
	**A**	**B**	**C**	**D**	
No. of samples	29	28	22	20	99
ER+ (ER−)	28 (1)	27 (1)	7 (15)	5 (15)	67 (32)
PR+ (PR−)	19 (8)	24 (3)	2 (20)	3 (17)	48 (48)*
HER2 IHC 3+ (2+)	16 (13)	23 (5)	20 (2)	16 (4)	75 (24)
					
PAM50					
Luminal A	2	1	0	0	3
Luminal B	27	23	4	1	55
Her2-enriched	0	4	18	13	35
Basal	0	0	0	6	6
Whole-genome seq.	21	17	12	14	64

^*^Three cases with unknown PR status.

## References

[b1] Sørlie T. *et al.* Gene expression patterns of breast carcinomas distinguish tumor subclasses with clinical implications. Proc. Natl Acad. Sci. USA 98, 10869–10874 (2001). 11553815 10.1073/pnas.191367098 PMC58566

[b2] Prat A. *et al.* Molecular features and survival outcomes of the intrinsic subtypes within HER2-positive breast cancer. J. Natl Cancer Inst. 106, dju152 (2014). 25139534 10.1093/jnci/dju152 PMC4151853

[b3] Slamon D. J. *et al.* Use of chemotherapy plus a monoclonal antibody against HER2 for metastatic breast cancer that overexpresses HER2. N. Engl. J. Med. 344, 783–792 (2001). 11248153 10.1056/NEJM200103153441101

[b4] Marty M. *et al.* Randomized phase II trial of the efficacy and safety of trastuzumab combined with docetaxel in patients with human epidermal growth factor receptor 2-positive metastatic breast cancer administered as first-line treatment: the M77001 study group. J. Clin. Oncol. 23, 4265–4274 (2005). 15911866 10.1200/JCO.2005.04.173

[b5] Romond E. H. *et al.* Trastuzumab plus adjuvant chemotherapy for operable HER2-positive breast cancer. N. Engl. J. Med. 353, 1673–1684 (2005). 16236738 10.1056/NEJMoa052122

[b6] Piccart-Gebhart M. J. *et al.* Trastuzumab after adjuvant chemotherapy in HER2-positive breast cancer. N. Engl. J. Med. 353, 1659–1672 (2005). 16236737 10.1056/NEJMoa052306

[b7] Piccart-Gebhart M. *et al.* Adjuvant lapatinib and trastuzumab for early human epidermal growth factor receptor 2-positive breast cancer: results from the randomized phase iii adjuvant lapatinib and/or trastuzumab treatment optimization trial. J. Clin. Oncol. 34, 1034–1042 (2014). 26598744 10.1200/JCO.2015.62.1797 PMC4872016

[b8] von Minckwitz G. *et al.* Definition and impact of pathologic complete response on prognosis after neoadjuvant chemotherapy in various intrinsic breast cancer subtypes. J. Clin. Oncol. 30, 1796–1804 (2012). 22508812 10.1200/JCO.2011.38.8595

[b9] Denkert C. *et al.* HER2 and ESR1 mRNA expression levels and response to neoadjuvant trastuzumab plus chemotherapy in patients with primary breast cancer. Breast Cancer Res. 15, R11 (2013). 23391338 10.1186/bcr3384 PMC3672694

[b10] Cortazar P. *et al.* Pathological complete response and long-term clinicalbenefit in breast cancer: the CTNeoBC pooled analysis. Lancet 384, 164–172 (2014). 24529560 10.1016/S0140-6736(13)62422-8

[b11] Carey L. A. *et al.* Molecular heterogeneity and response to neoadjuvant human epidermal growth factor receptor 2 targeting in CALGB 40601, a randomized phase III trial of paclitaxel plus trastuzumab with or without lapatinib. J. Clin. Oncol. 34, 542–549 (2015). 26527775 10.1200/JCO.2015.62.1268 PMC4980567

[b12] Esteva F. J. *et al.* PTEN, PIK3CA, p-AKT, and p-p70S6K status: association with trastuzumab response and survival in patients with HER2-positive metastatic breast cancer. Am. J. Pathol. 177, 1647–1656 (2010). 20813970 10.2353/ajpath.2010.090885 PMC2947262

[b13] Jensen J. D. *et al.* PIK3CA mutations, PTEN, and pHER2 expression and impact on outcome in HER2-positive early-stage breast cancer patients treated with adjuvant chemotherapy and trastuzumab. Ann. Oncol. 23, 2034–2042 (2012). 22172323 10.1093/annonc/mdr546

[b14] Baselga J. *et al.* Biomarker analyses in CLEOPATRA: a phase III, placebo-controlled study of pertuzumab in human epidermal growth factor receptor 2-positive, first-line metastatic breast cancer. J. Clin. Oncol. 32, 3753–3761 (2014). 25332247 10.1200/JCO.2013.54.5384

[b15] Loibl S. *et al.* PIK3CA mutations are associated with lower rates of pathologic complete response to anti-human epidermal growth factor receptor 2 (her2) therapy in primary HER2-overexpressing breast cancer. J. Clin. Oncol. 32, 3212–3220 (2014). 25199759 10.1200/JCO.2014.55.7876

[b16] Majewski I. J. *et al.* PIK3CA mutations are associated with decreased benefit to neoadjuvant human epidermal growth factor receptor 2-targeted therapies in breast cancer. J. Clin. Oncol. 33, 1334–1339 (2015). 25559818 10.1200/JCO.2014.55.2158 PMC5087318

[b17] Rody A. *et al.* T-cell metagene predicts a favorable prognosis in estrogen receptor-negative and HER2-positive breast cancers. Breast Cancer Res. 11, R15 (2009). 19272155 10.1186/bcr2234 PMC2688939

[b18] Bianchini G. *et al.* Immune modulation of pathologic complete response after neoadjuvant HER2-directed therapies in the NeoSphere trial. Ann. Oncol. 26, 2429–2436 (2015). 26387142 10.1093/annonc/mdv395

[b19] Pivot X., Romieu G., Debled M., Pierga J.-Y. & Kerbrat P. 6 months versus 12 months of adjuvant trastuzumab for patients with HER2-positive early breast cancer (PHARE): a randomised phase 3 trial. Lancet Oncol. 14, 741–748 (2013). 23764181 10.1016/S1470-2045(13)70225-0

[b20] Kramar A. *et al.* Trastuzumab duration effects within patient prognostic subgroups in the PHARE trial. Ann. Oncol. 25, 1563–1570 (2014). 24827132 10.1093/annonc/mdu177

[b21] Wolff A. C. *et al.* American Society of Clinical Oncology/College of American Pathologists guideline recommendations for human epidermal growth factor receptor 2 testing in breast cancer. J. Clin. Oncol. 25, 118–145 (2007). 17159189 10.1200/JCO.2006.09.2775

[b22] Cancer Genome Atlas Network. Comprehensive molecular portraits of human breast tumours. Nature 490, 61–70 (2012). 23000897 10.1038/nature11412 PMC3465532

[b23] Curtis C. *et al.* Supplemental information: the genomic and transcriptomic architecture of 2,000 breast tumours reveals novel subgroups. Nature 486, 346–352 (2012). 22522925 10.1038/nature10983 PMC3440846

[b24] Parker J. S. *et al.* Supervised risk predictor of breast cancer based on intrinsic subtypes. J. Clin. Oncol. 27, 1160–1167 (2009). 19204204 10.1200/JCO.2008.18.1370 PMC2667820

[b25] Guedj M. *et al.* A refined molecular taxonomy of breast cancer. Oncogene 31, 1196–1206 (2011). 21785460 10.1038/onc.2011.301 PMC3307061

[b26] Lim E. *et al.* Aberrant luminal progenitors as the candidate target population for basal tumor development in BRCA1 mutation carriers. Nat. Med. 15, 907–913 (2009). 19648928 10.1038/nm.2000

[b27] Stephens P. J. *et al.* The landscape of cancer genes and mutational processes in breast cancer. Nature 486, 400–404 (2012). 22722201 10.1038/nature11017 PMC3428862

[b28] Nik-Zainal S. *et al.* Mutational processes molding the genomes of 21 breast cancers. Cell 149, 979–993 (2012). 22608084 10.1016/j.cell.2012.04.024 PMC3414841

[b29] Langerød A. *et al.* TP53 mutation status and gene expression profiles are powerful prognostic markers of breast cancer. Breast Cancer Res. 9, R30 (2007). 17504517 10.1186/bcr1675 PMC1929092

[b30] Silwal-Pandit L. *et al.* TP53 mutation spectrum in breast cancer is subtype specific and has distinct prognostic relevance. Clin. Cancer Res. 20, 3569–3580 (2014). 24803582 10.1158/1078-0432.CCR-13-2943

[b31] Kancha R. K. *et al.* Differential sensitivity of ERBB2 kinase domain mutations towards lapatinib. PLoS ONE 6, e26760 (2011). 22046346 10.1371/journal.pone.0026760 PMC3203921

[b32] Perera D. *et al.* OncoCis: annotation of cis-regulatory mutations in cancer. Genome Biol. 15, 485 (2014). 25298093 10.1186/s13059-014-0485-0 PMC4224696

[b33] Staaf J. *et al.* High-resolution genomic and expression analyses of copy number alterations in HER2-amplified breast cancer. Breast Cancer Res. 12, R25 (2010). 20459607 10.1186/bcr2568 PMC2917012

[b34] Sircoulomb F. *et al.* Genome profiling of ERBB2-amplified breast cancers. BMC Cancer 10, 539 (2010). 20932292 10.1186/1471-2407-10-539 PMC2958950

[b35] Hicks J. *et al.* Novel patterns of genome rearrangement and their association with survival in breast cancer. Genome Res. 16, 1465–1479 (2006). 17142309 10.1101/gr.5460106 PMC1665631

[b36] Yang L. *et al.* Diverse mechanisms of somatic structural variations in human cancer genomes. Cell 153, 919–929 (2013). 23663786 10.1016/j.cell.2013.04.010 PMC3704973

[b37] Popova T. *et al.* Ploidy and large-scale genomic instability consistently identify basal-like breast carcinomas with BRCA1/2 inactivation. Cancer Res. 72, 5454–5462 (2012). 22933060 10.1158/0008-5472.CAN-12-1470

[b38] McClintock B. The stability of broken ends of chromosomes in Zea Mays. Genetics 26, 234–282 (1941). 17247004 10.1093/genetics/26.2.234 PMC1209127

[b39] Bignell G. R. *et al.* Architectures of somatic genomic rearrangement in human cancer amplicons at sequence-level resolution. Genome Res. 17, 1296–1303 (2007). 17675364 10.1101/gr.6522707 PMC1950898

[b40] Campbell P. J. *et al.* The patterns and dynamics of genomic instability in metastatic pancreatic cancer. Nature 467, 1109–1113 (2010). 20981101 10.1038/nature09460 PMC3137369

[b41] Marotta M. *et al.* A common copy-number breakpoint of ERBB2 amplification in breast cancer colocalizes with a complex block of segmental duplications. Breast Cancer Res. 14, R150 (2012). 23181561 10.1186/bcr3362 PMC4053137

[b42] Zakov S., Kinsella M. & Bafna V. An algorithmic approach for breakage-fusion-bridge detection in tumor genomes. Proc. Natl Acad. Sci. USA 110, 5546–5551 (2013). 23503850 10.1073/pnas.1220977110 PMC3619374

[b43] Greenman C. D., Cooke S. L., Marshall J., Stratton M. R. & Campbell P. J. Modeling the evolution space of breakage fusion bridge cycles with a stochastic folding process. J. Math. Biol. 72, 47–86 (2015). 25833184 10.1007/s00285-015-0875-2 PMC4702116

[b44] Sanborn J. Z. *et al.* Double minute chromosomes in glioblastoma multiforme are revealed by precise reconstruction of oncogenic amplicons. Cancer Res. 73, 6036–6045 (2013). 23940299 10.1158/0008-5472.CAN-13-0186 PMC3800429

[b45] Bolstad B. M. *et al.* in Bioinformatics and Computational Biology Solutions using R and Bioconductor eds Gentleman R., Carey V., Huber W., Irizarry R. A., Dudoit S. 33–47Springer (2005).

[b46] Bolstad B. M., Irizarry R. A., Astrand M. & Speed T. P. A comparison of normalization methods for high density oligonucleotide array data based on variance and bias. Bioinformatics 19, 185–193 (2003). 12538238 10.1093/bioinformatics/19.2.185

[b47] Gautier L., Cope L., Bolstad B. M. & Irizarry R. A. affy--analysis of Affymetrix GeneChip data at the probe level. Bioinformatics 20, 307–315 (2004). 14960456 10.1093/bioinformatics/btg405

[b48] Sørlie T. *et al.* The importance of gene-centring microarray data. Lancet Oncol. 11, 719–720 (2010). 20688275 10.1016/S1470-2045(10)70174-1

[b49] Gendoo D. M. *et al.* genefu: Computation of Gene Expression-Based Signatures in Breast Cancer. R package version 2.5.2. Available at http://www.pmgenomics.ca/bhklab/software/genefu (2015). 10.1093/bioinformatics/btv693 PMC6410906 26607490

[b50] Simon R. & Lam A. BRB Array Tools Users Guide. Technical Reports. Biometric Research Branch, National Cancer Institute. Available at http://linus.nci.nih.gov/brb/TechReport (2006).

[b51] Dunning M. J., Smith M. L., Ritchie M. E. & Tavaré S. beadarray: R classes and methods for Illumina bead-based data. Bioinformatics 23, 2183–2184 (2007). 17586828 10.1093/bioinformatics/btm311

[b52] Li Q., Birkbak N. J., Gyorffy B., Szallasi Z. & Eklund A. C. Jetset: selecting the optimal microarray probe set to represent a gene. BMC Bioinformatics 12, 474 (2011). 22172014 10.1186/1471-2105-12-474 PMC3266307

[b53] Barbie D. A. *et al.* Systematic RNA interference reveals that oncogenic KRAS-driven cancers require TBK1. Nature 462, 108–112 (2009). 19847166 10.1038/nature08460 PMC2783335

[b54] Staaf J. *et al.* Normalization of Illumina Infinium whole-genome SNP data improves copy number estimates and allelic intensity ratios. BMC Bioinformatics 9, 409 (2008). 18831757 10.1186/1471-2105-9-409 PMC2572624

[b55] Popova T. *et al.* Genome alteration print (GAP): a tool to visualize and mine complex cancer genomic profiles obtained by SNP arrays. Genome Biol. 10, R128 (2009). 19903341 10.1186/gb-2009-10-11-r128 PMC2810663

[b56] Van Loo P. *et al.* Analyzing cancer samples with SNP arrays. Methods Mol. Biol. 802, 57–72 (2012). 22130873 10.1007/978-1-61779-400-1_4

[b57] Li H. & Durbin R. Fast and accurate short read alignment with Burrows-Wheeler transform. Bioinformatics 25, 1754–1760 (2009). 19451168 10.1093/bioinformatics/btp324 PMC2705234

[b58] McKenna A. *et al.* The Genome Analysis Toolkit: a MapReduce framework for analyzing next-generation DNA sequencing data. Genome Res. 20, 1297–1303 (2010). 20644199 10.1101/gr.107524.110 PMC2928508

[b59] Cibulskis K. *et al.* Sensitive detection of somatic point mutations in impure and heterogeneous cancer samples. Nat. Biotechnol. 31, 213–219 (2013). 23396013 10.1038/nbt.2514 PMC3833702

[b60] McLaren W. *et al.* Deriving the consequences of genomic variants with the Ensembl API and SNP effect predictor. Bioinformatics 26, 2069–2070 (2010). 20562413 10.1093/bioinformatics/btq330 PMC2916720

[b61] Andersson R. *et al.* An atlas of active enhancers across human cell types and tissues. Nature 507, 455–461 (2014). 24670763 10.1038/nature12787 PMC5215096

[b62] Benjamini Y. & Speed T. P. Summarizing and correcting the GC content bias in high-throughput sequencing. Nucleic Acids Res. 40, e72 (2012). 22323520 10.1093/nar/gks001 PMC3378858

[b63] Derrien T. *et al.* Fast computation and applications of genome mappability. PLoS ONE 7, e30377 (2012). 22276185 10.1371/journal.pone.0030377 PMC3261895

[b64] Zakov S. & Bafna V. Reconstructing breakage fusion bridge architectures using noisy copy numbers. J. Comput. Biol. 22, 577–594 (2015). 26020441 10.1089/cmb.2014.0166 PMC4449712

[b65] Greenman C. D. *et al.* Estimation of rearrangement phylogeny for cancer genomes. Genome Res. 22, 346–361 (2012). 21994251 10.1101/gr.118414.110 PMC3266042

[b66] Greenacre M. & Blasius J. Multiple Correspondence Analysis and Related Methods Chapman and Hall/CRC (2006).

[b67] Thioulouse J., Chessel D., Dolédec S. & Olivier J. M. ADE-4: a multivariate analysis and graphical display software. Stat. Comput. 7, 75–83 (1997).

[b68] Benzécri J. P. Sur le calcul des taux d'inertie dans l'analyse d'un questionnaire. Cahiers de l'Analyse des Données 4, 377–378 (1979).

[b69] Hoffman D. L. & De Leeuw J. Interpreting multiple correspondence analysis as a multidimensional scaling method. Mark, Lett. 3, 259–272 (1992).

