# Supplementary material for: A whole-genome sequence and transcriptome perspective on HER2-positive breast cancers
Source: Nat Commun. 2016 Jul 13;7:12222. doi: 10.1038/ncomms12222 (PMC4947184; doi:10.1038/ncomms12222)
Supplement: Supplementary Information — Supplementary Figures 1-17 [file ncomms12222-s1.pdf]

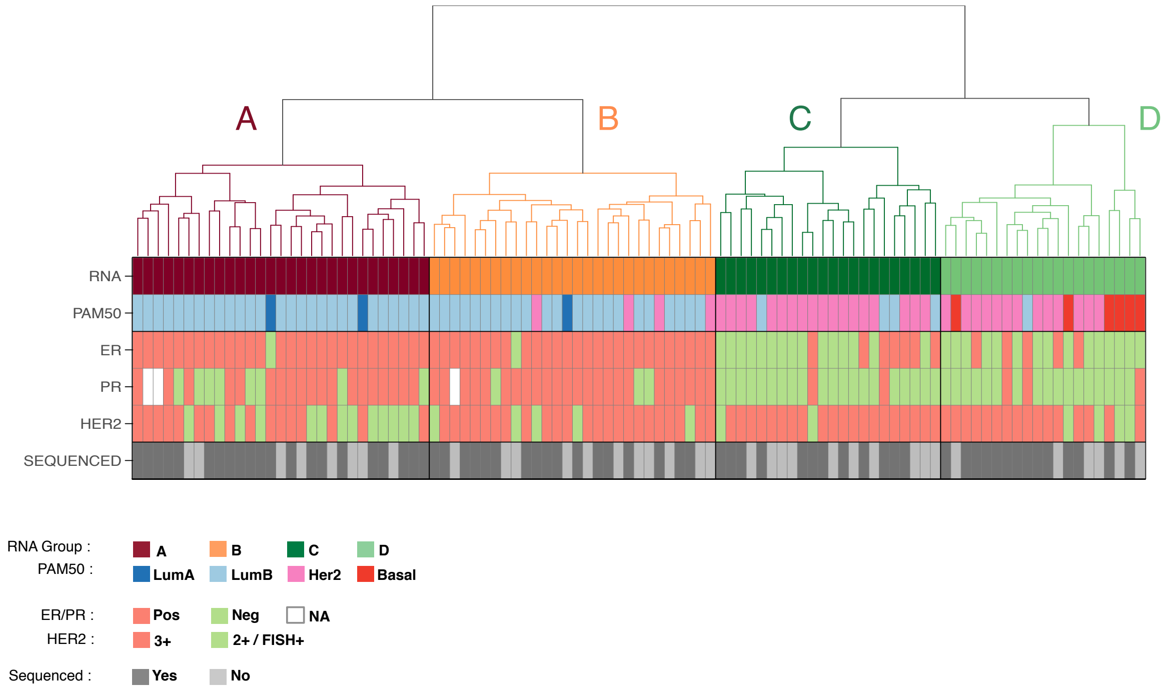

Supplementary Figure 1: Unsupervised hierarchical clustering of transcriptomic array profiles of the 99 HER2+ samples. Four RNA expression groups, named A, B, C and D, were delineated (Methods) and respectively coloured in red, orange, dark green and light green. **Lower panel:** PAM50 subtypes; ER, PR and HER2 IHC statuses; last line indicates the 64 sequenced samples.

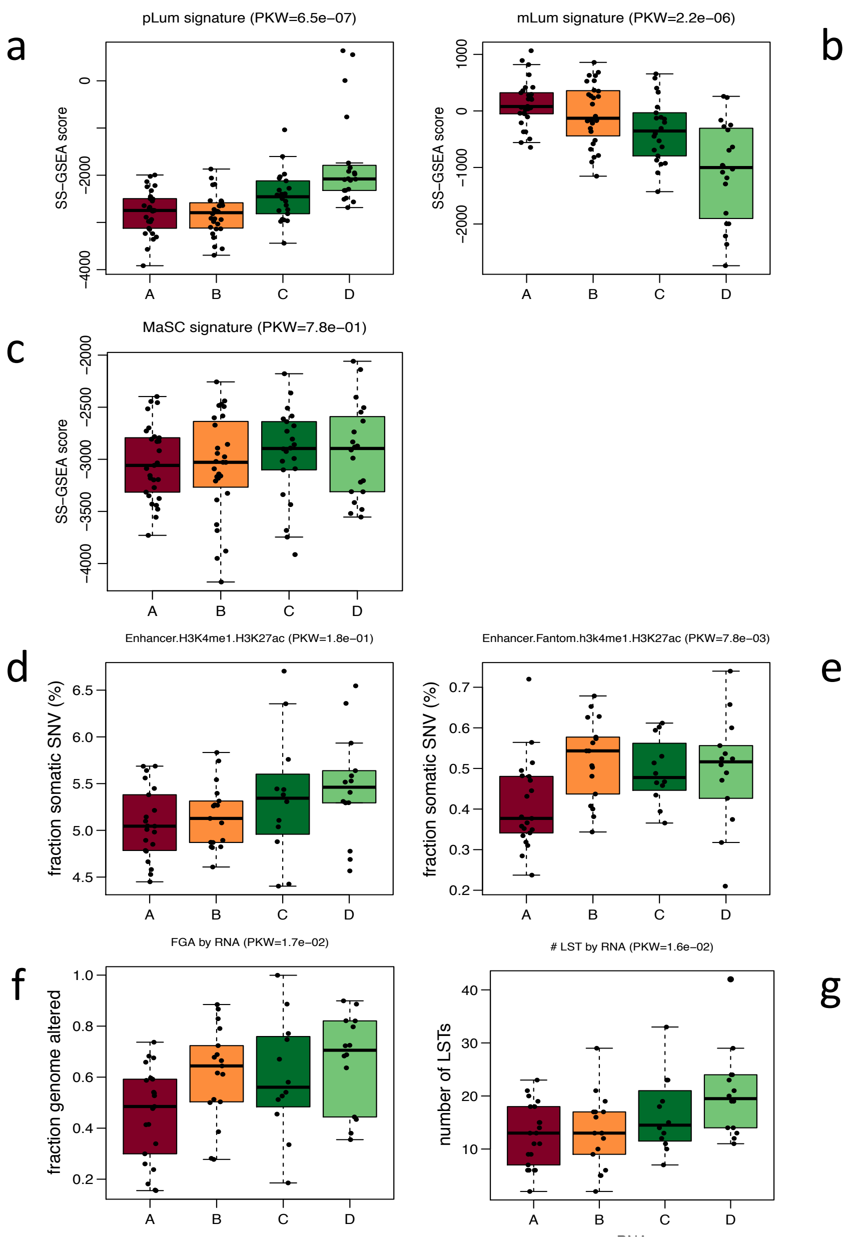

Supplementary Figure 2 a-b-c-d-e-f-g: Distribution of several markers by RNA expression groups. Expression profile (SS-GSEA score) of pLum **(a)**, mLum **(b)** and MaSC **(c)** specific gene sets (Methods). Fraction of somatic SNVs located in enhancers regions: H3K4me1, H3K27ac **(d)** H3K4me1, H3K27ac + Fantom5 **(e)**. **(f)** Fraction of genome altered (FGA). **(g)** Number of Large Scale Transitions (BRCAness score). Boxes indicate the interquartile range (IQR); center line, median; whiskers, lowest and highest values within 1.5× IQR from the first and third quartiles, respectively.

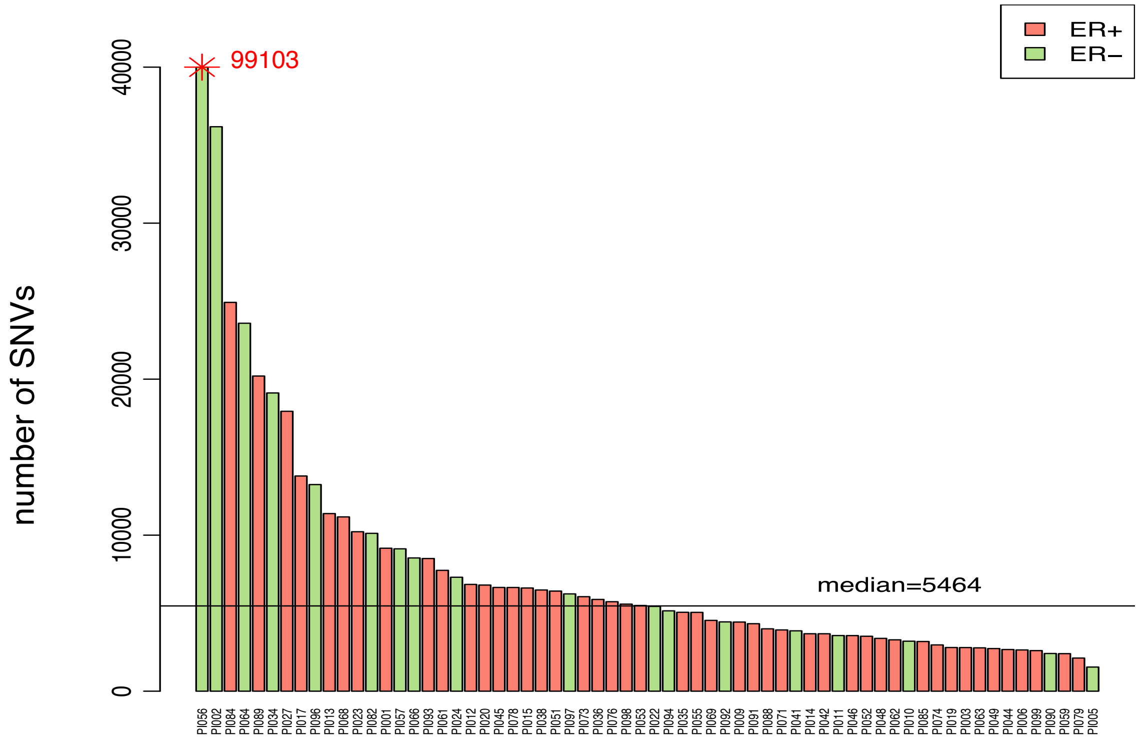

Supplementary Figure 3: Distribution of the number of somatic SNVs in the 64 sequenced HER2+ samples. ER status of each sample is represented by red (ER+) and green (ER-) bars.

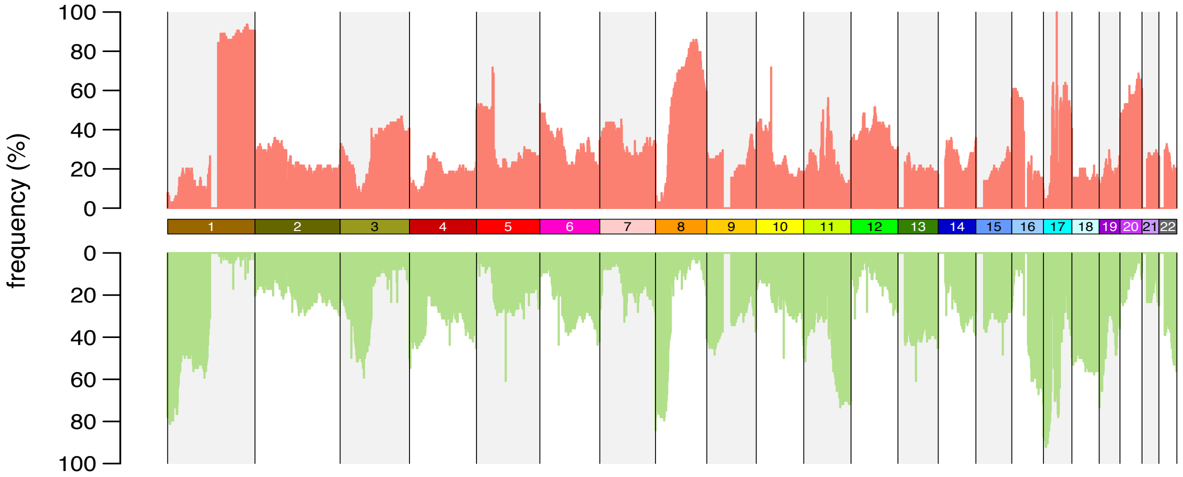

Supplementary Figure 4: Frequency plots of gain and loss in the 64 sequenced HER2+ samples. Gain (red) and loss (green) relative frequencies (% tumours) were computed per 10kb segments from the somatic copy number alterations (CNVs) data. Chromosome names are displayed as coloured rectangles in the middle row.

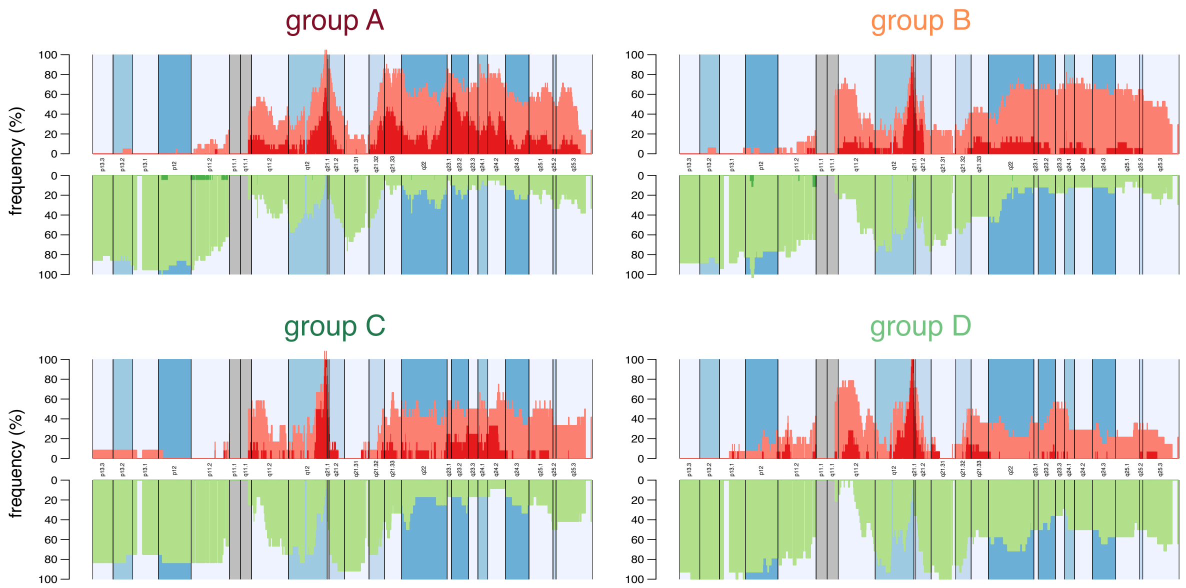

Supplementary Figure 5: Amplified regions on chromosome 17. Amplification (red), gain (pink), loss (light green) and homozygous loss (dark green) relative frequencies (% of tumours) on chromosome 17 per 10 kb segments. The frequency plots are given for each RNA expression group (A to D) separately.

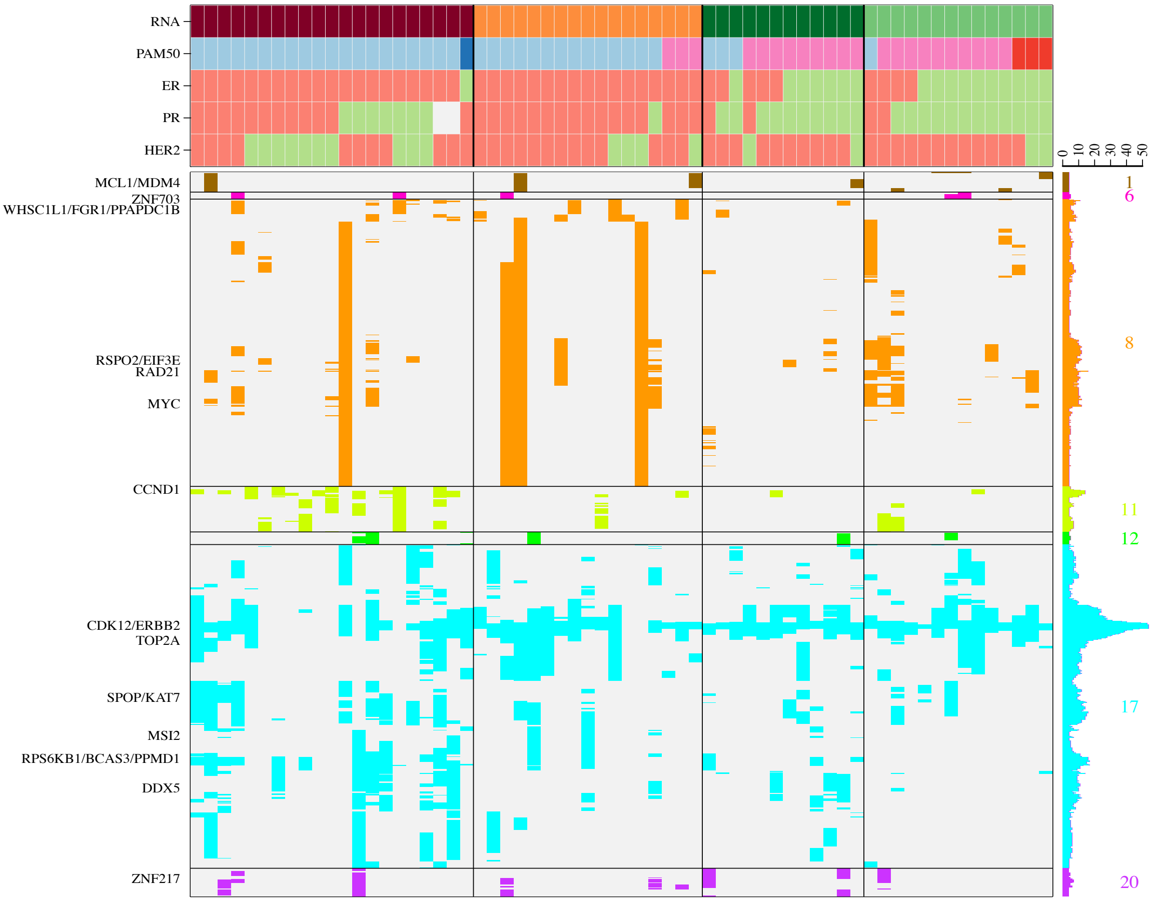

Supplementary Figure 6: Summary of amplified genes in the 64 sequenced HER2+ samples. Upper panel: from top to bottom : RNA expression groups, PAM50 subtypes, ER, PR and HER2 IHC statuses (same colour scheme as in Supplementary Fig. 1); **Lower panel:** amplified genes. Colours are associated to chromosomes as indicated in the right part of the figure. Genes positions (on each chromosome) are increasing from top to bottom. Some genes of interest are labelled on the left part of the figure.

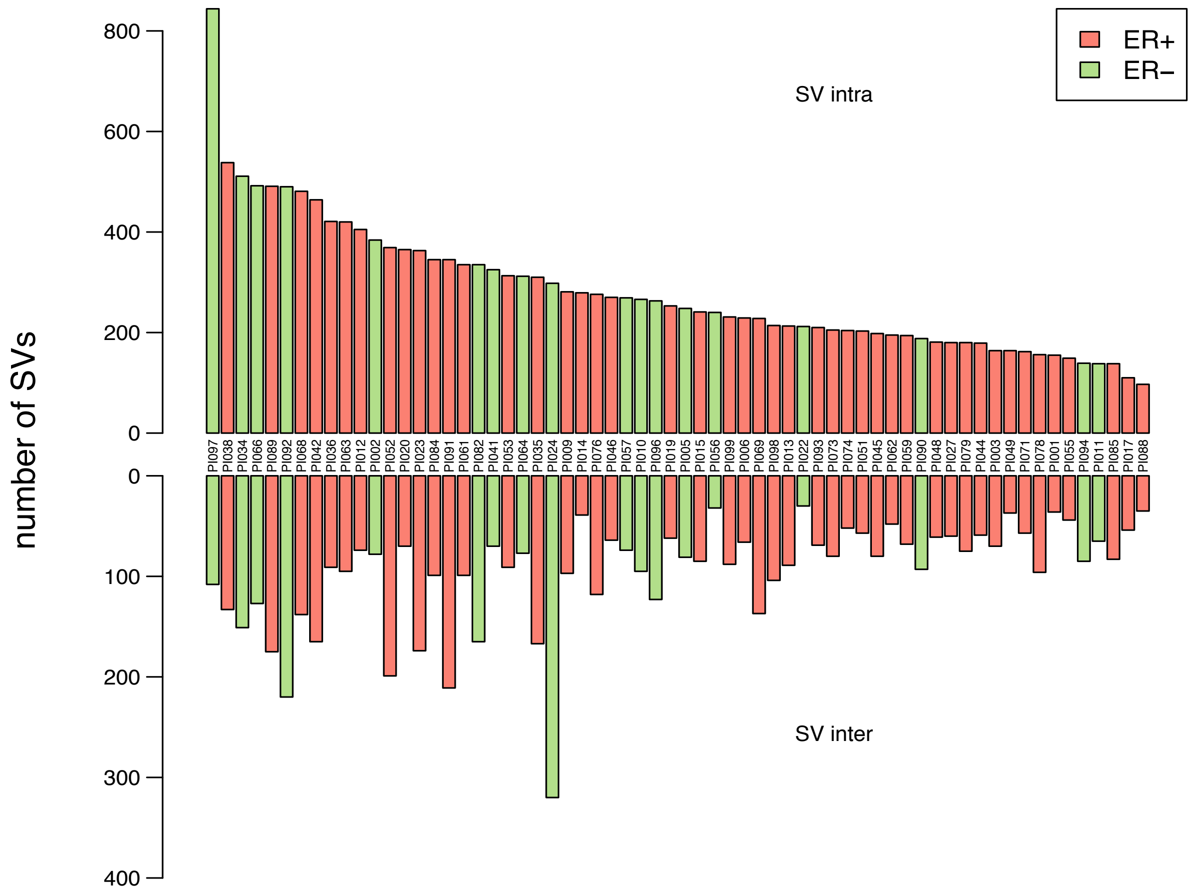

Supplementary Figure 7: Distribution of the number of somatic SVs in the 64 sequenced HER2+ samples. SVs were stratified into intra-chromosomal (upper part) and inter-chromosomal (lower part).

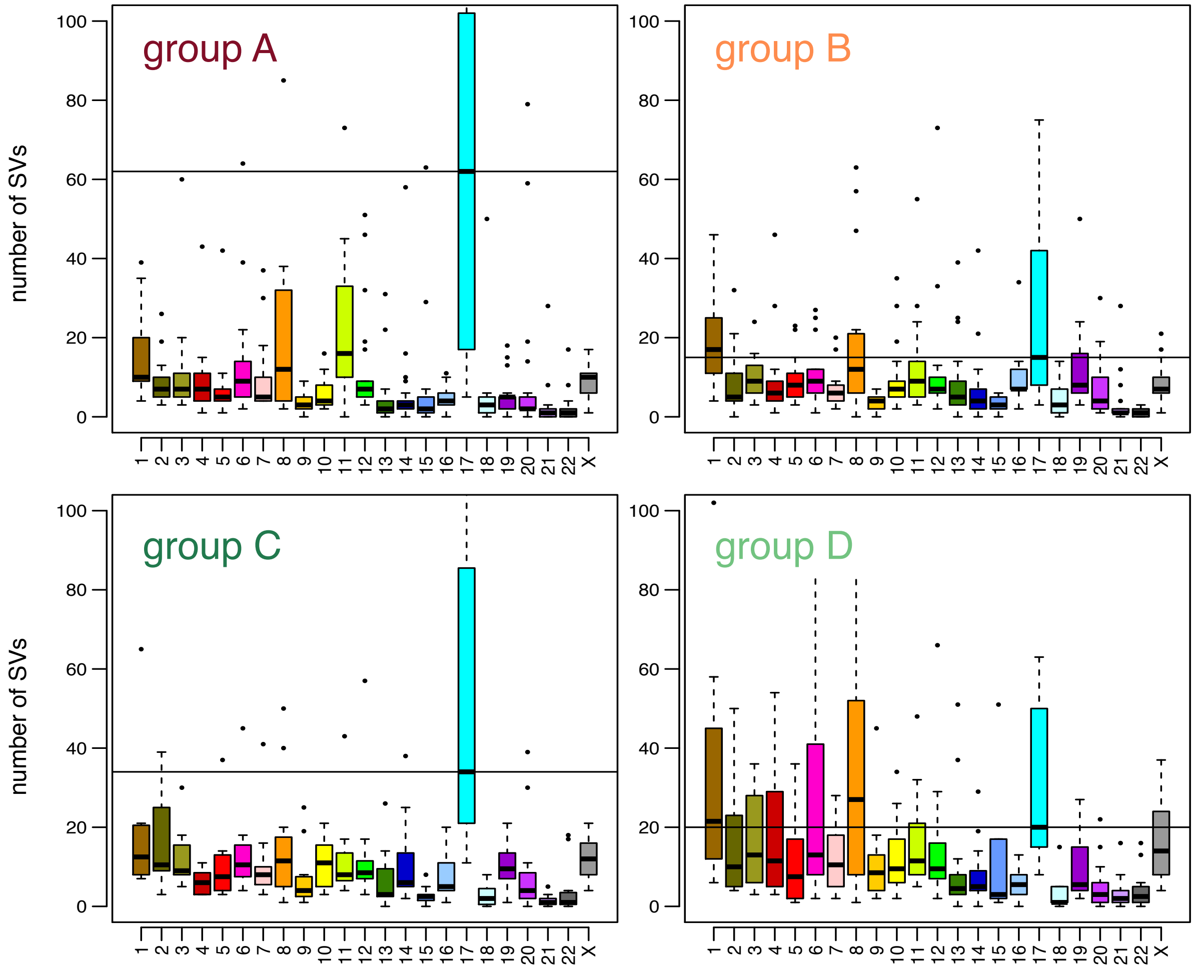

Supplementary Figure 8: Distributions of the number of intra-chromosomal somatic SVs per chromosome stratified by RNA expression group. X axis: chromosome; Y axis distribution of the number of intra-chromosomal somatic SVs represented by a box plot (interquartile range).

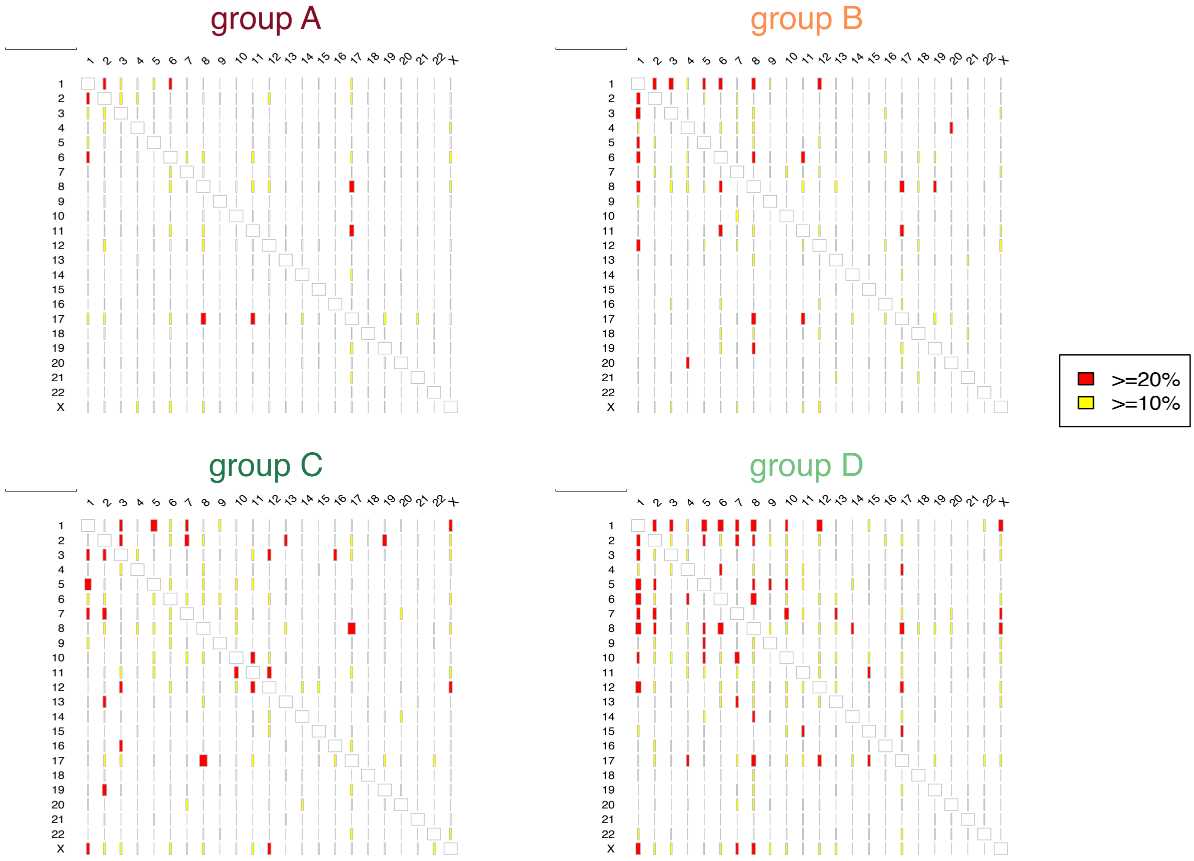

Supplementary Figure 9: 2D-frequency plots of inter-chromosomal somatic SVs stratified by RNA expression group. Each entry of the plotted matrix represents the percentage of samples harbouring at least 2 somatic SVs between the corresponding pair of chromosomes. Boxes on the diagonal indicate the 100% scale. Red boxes indicate values ≥ 20% and yellow boxes indicate values ≥ 10%.

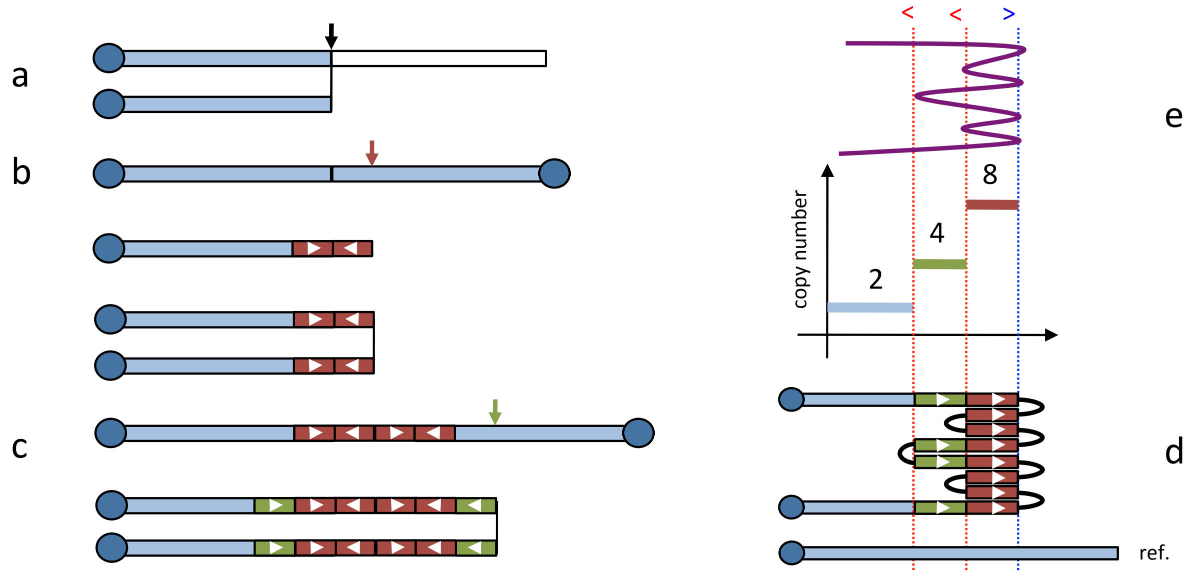

Supplementary Figure 10 : Schematic BFB process and NGS landmarks.

(a) Initial double-strand break (black arrow), followed by fusion of sister chromatids (vertical line) and formation of a dicentric chromosome (b) that will further break at the next cell cycle (red arrow). Centromeres are represented as dark blue circles and the process is depicted here on the q arm. (c) second BFB cycle. Coloured stretches indicate identical sequences and white arrows indicate their orientation. Copy number and fold-back patterns generated by these two BFB cycles are displayed in (d) and (e). A red "<" (resp. blue ">") symbol indicates that reads spanning the breakpoint are clipped to the left (resp. to the right).

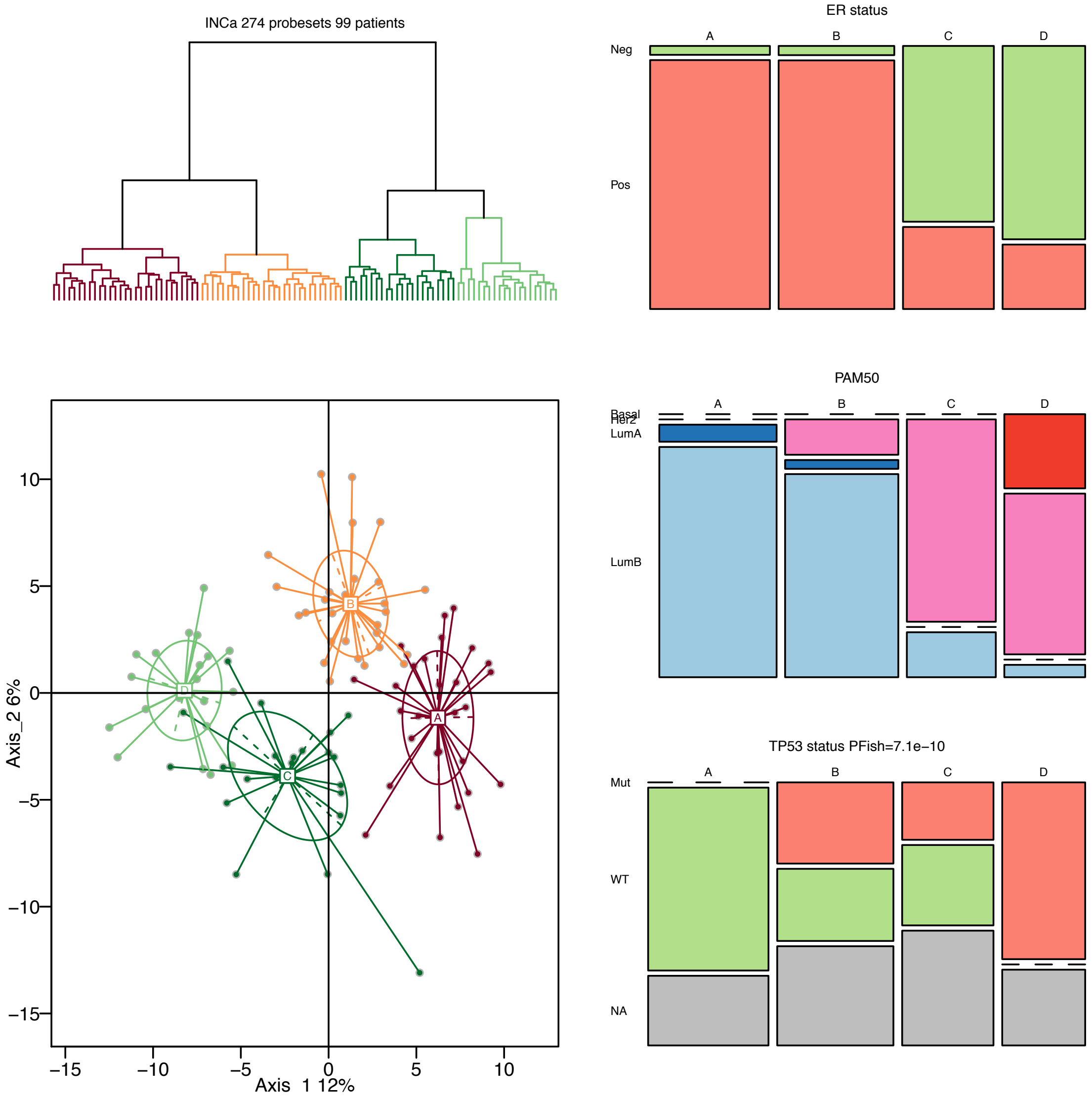

Supplementary Figure 11: Unsupervised clustering of INCa dataset transcriptomic array profiles. The tree (upper left corner) represents the 4 RNA expression groups detected: A, B, C and D coloured respectively in red, orange, dark green and light green. The lower left graph represents the same group with a PCA analysis. The ER status, PAM50 subtypes and *TP53* status (Mutated, Wildtype and NotAvailable) are detailed inside each group on the 3 right plots.

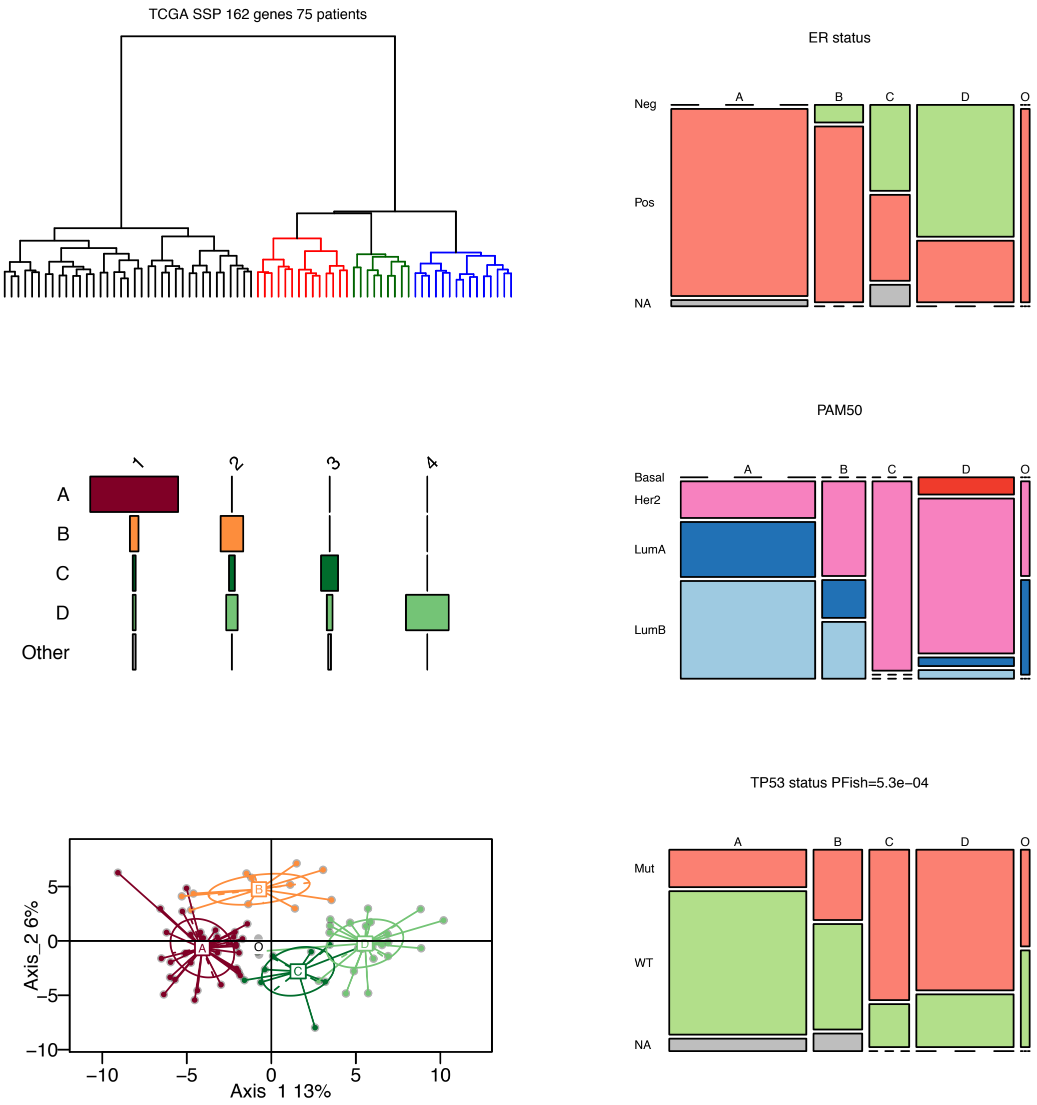

Supplementary Figure 12: Validation of the four RNA groups on the TCGA external dataset using the Single Sample Predictor (SSP) method. Each TCGA-HER2+ sample is first assigned to a RNA group (A, B, C, D or Other) using the SSP approach (Methods). All samples (n=75) are subjected to unsupervised clustering into four clusters (upper left tree) and RNA group frequencies in each cluster are depicted (middle left battleship plot). The mosaic plots on the right part display some selected features (ER status, PAM50 subtypes, *TP53* mutations) by assigned RNA groups.

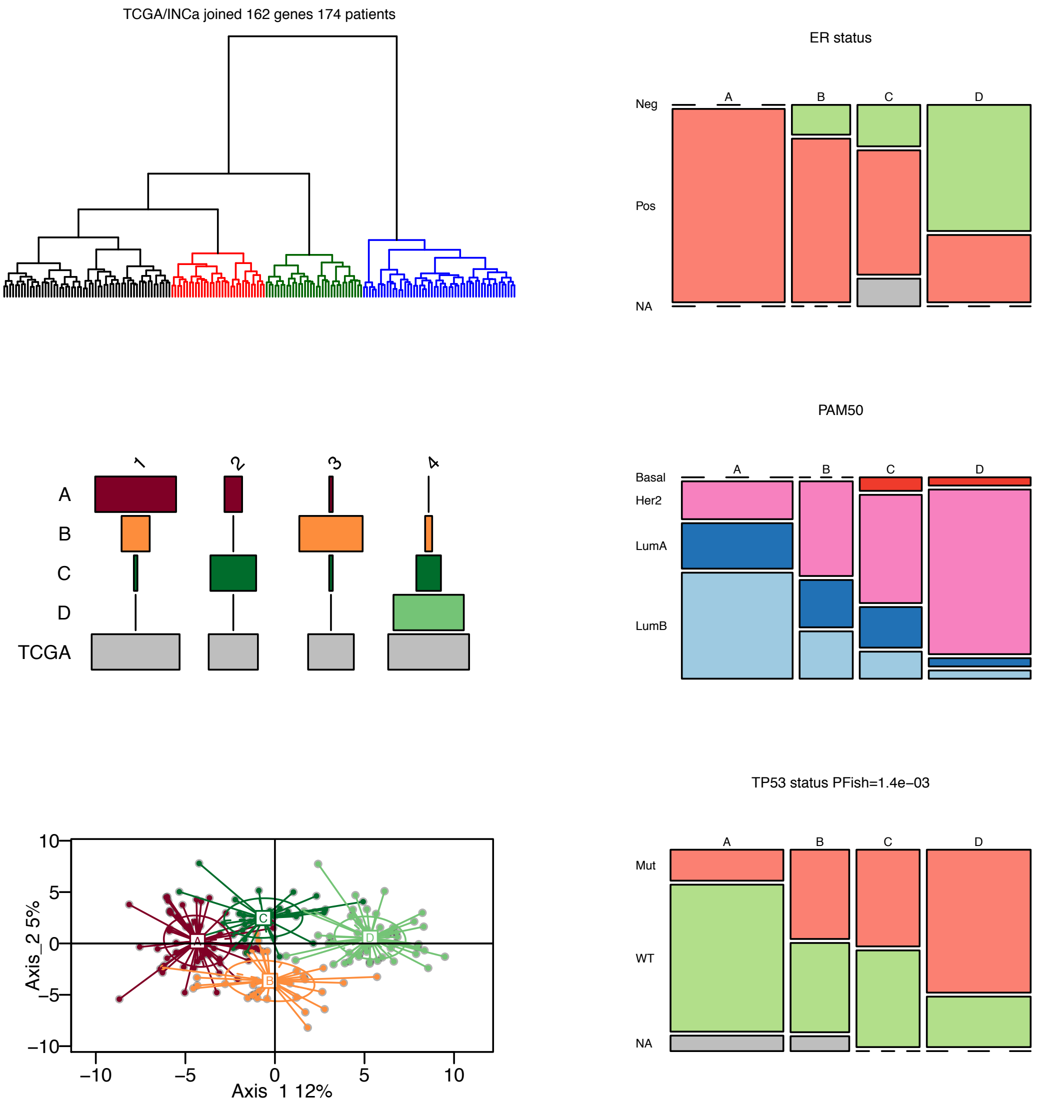

Supplementary Figure 13: Validation of the four RNA groups on the TCGA external dataset using the Joined method. TCGA-HER2+ (n=75) and INCa-HER2+ (n=99) samples are pooled and subjected to unsupervised clustering into four clusters (upper left tree). RNA group frequencies in each cluster for the INCa-HER2+ samples are depicted (middle left battleship plot). Each TCGA-HER2+ sample is then labelled with the most abundant RNA group in the cluster it belongs to (Methods). The mosaic plots on the right part display some selected features (ER status, PAM50 subtypes, *TP53* mutations) by assigned RNA groups.

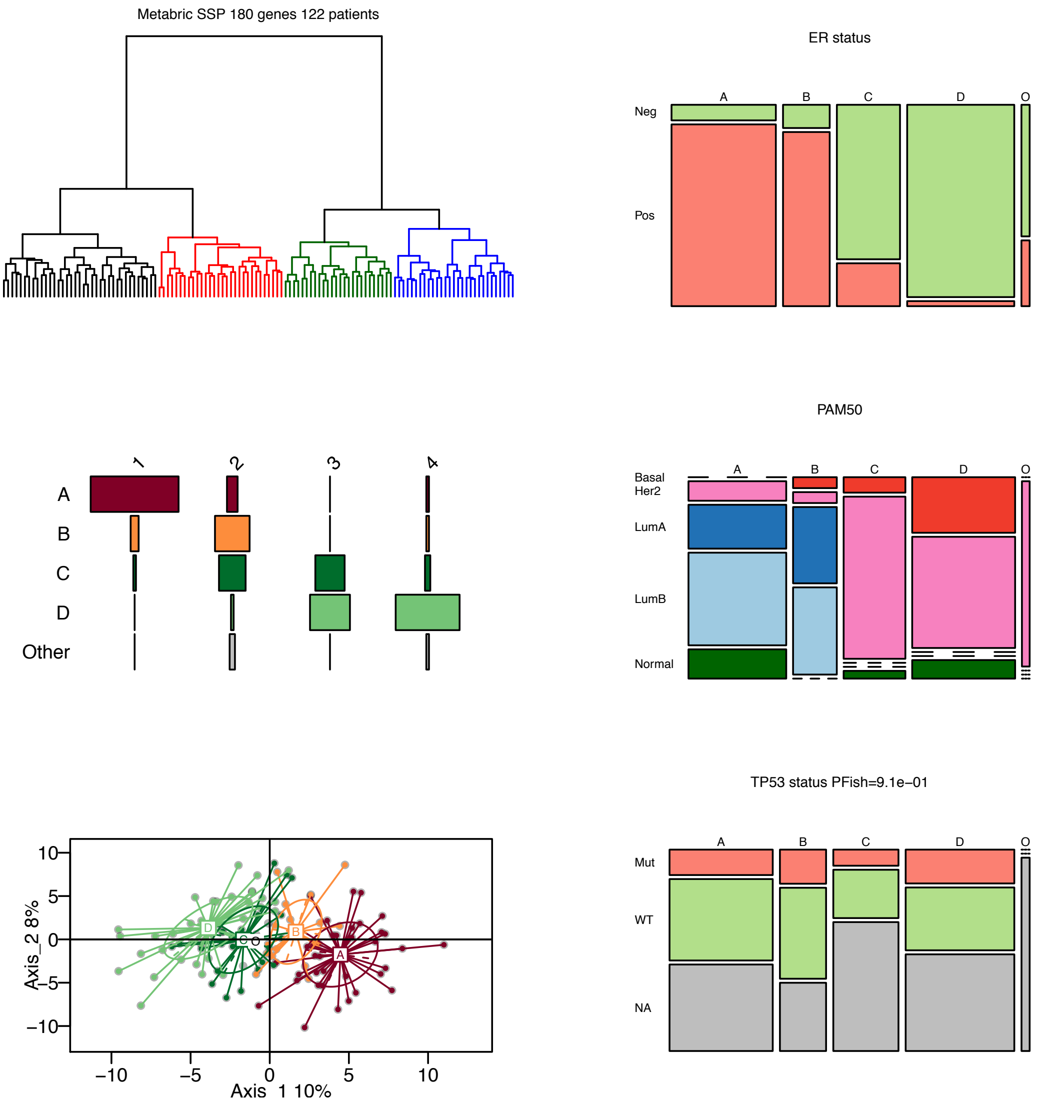

Supplementary Figure 14: Validation of the four RNA groups on the Metabric external dataset using the Single Sample Predictor (SSP) method. Each Metabric-HER2+ sample is first assigned to a RNA group (A, B, C, D or Other) using the SSP approach (Methods). All samples (n=122) are subjected to unsupervised clustering into four clusters (upper left tree) and the frequencies of RNA groups in each cluster are depicted (middle left battleship plot). The mosaic plots on the right part display some selected features (ER status, PAM50 subtypes, *TP53* mutations) by RNA groups.

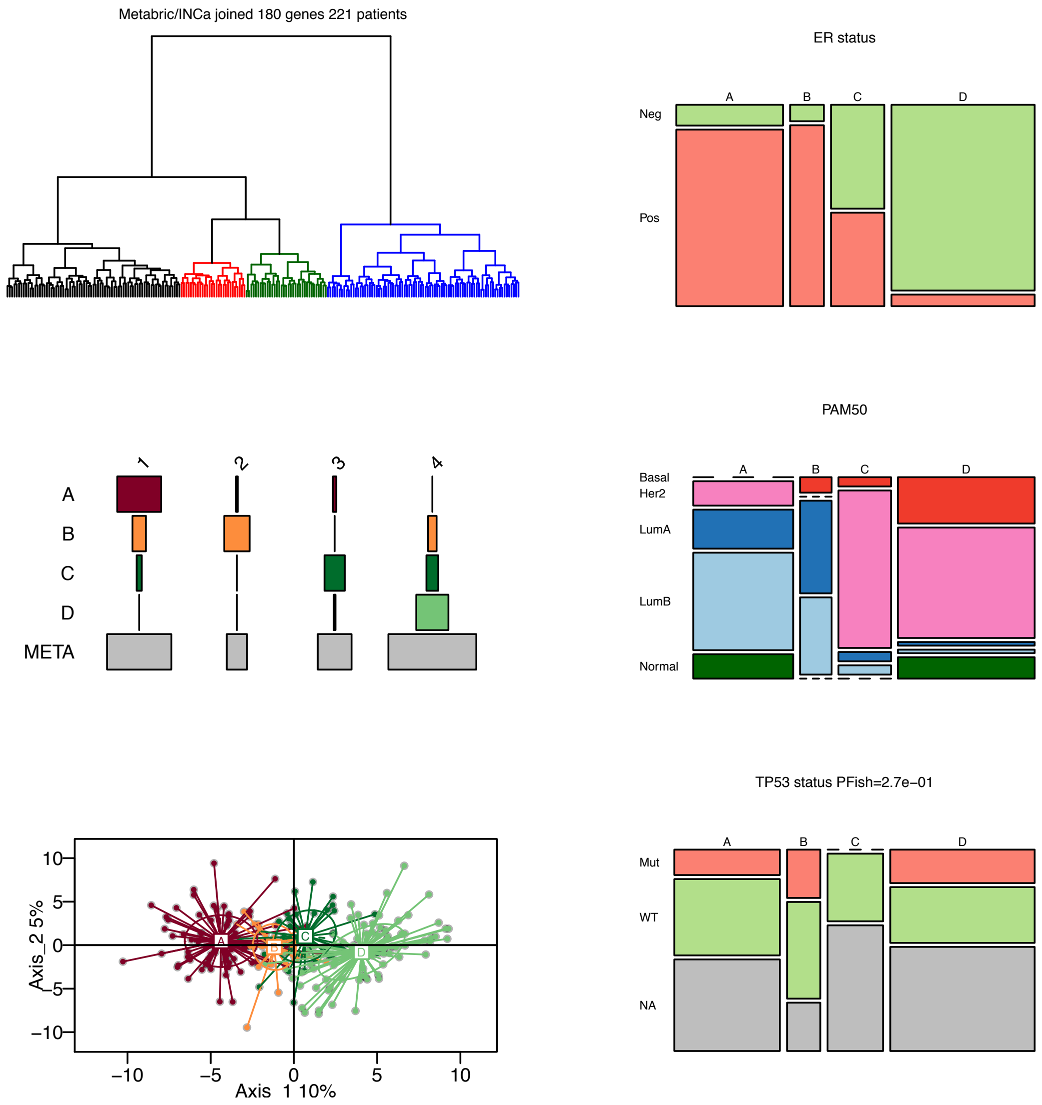

Supplementary Figure 15: Validation of the four RNA groups on the Metabric external dataset using the Joined method. Metabric-HER2+ (n=122) and INCa-HER2+ (n=99) samples are pooled and subjected to unsupervised clustering into four clusters (upper left tree). RNA group frequencies in each cluster for the INCa-HER2+ samples are depicted (middle left battleship plot). Each Metabric-HER2+ sample is then labelled with the most abundant RNA group in the cluster it belongs to (Methods). The mosaic plots on the right part display some selected features (ER status, PAM50 subtypes, *TP53* mutations) by assigned RNA groups.

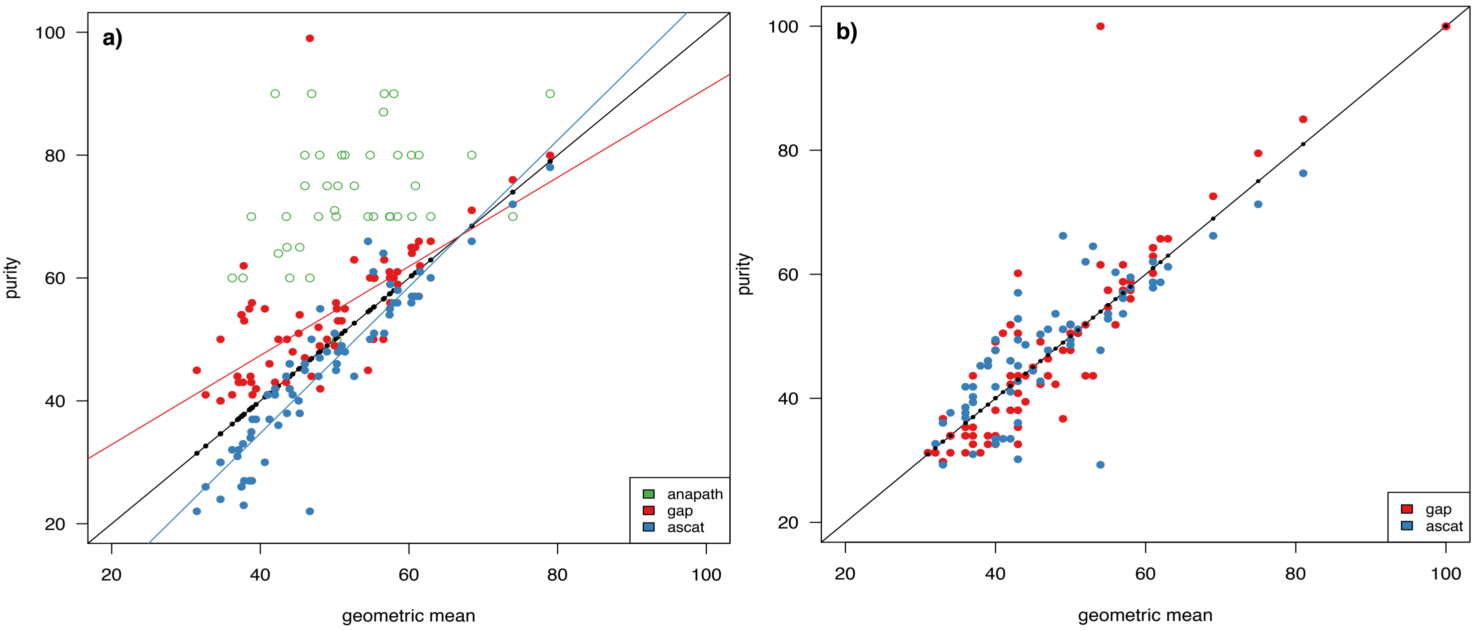

Supplementary Figure 16: Tumour purity estimations. Comparison of tumour DNA purity estimated by anatomo-pathologists (green circles), and by two algorithms array-based : GAP (red circles) and ASCAT (blue circles) algorithms. Raw values (a) and corrected values (b) are displayed.

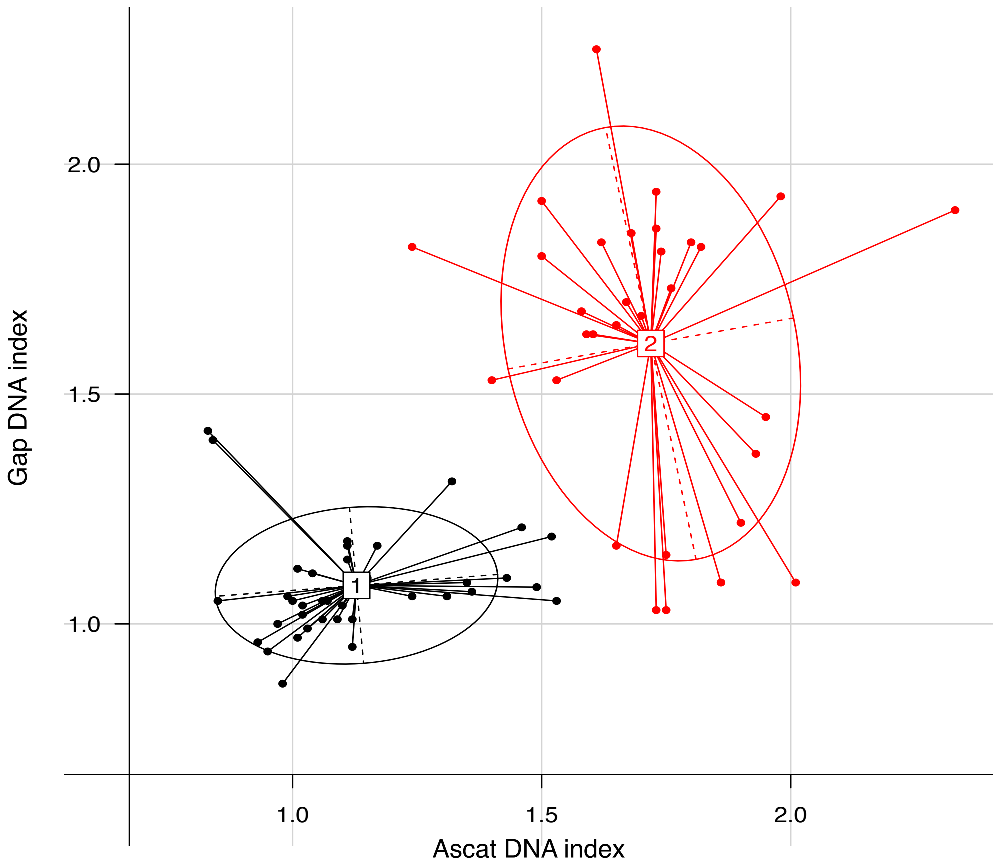

Supplementary Figure 17: Nearest neighbours clustering of ploidy values estimated by GAP and ASCAT. Group 1 corresponds to diploid tumours and group 2 corresponds to tetraploid tumours.
